# Dynamic Regulation of JAK-STAT Signaling Through the Prolactin Receptor Predicted by Computational Modeling

**DOI:** 10.1007/s12195-020-00647-8

**Published:** 2020-09-08

**Authors:** Ryland D. Mortlock, Senta K. Georgia, Stacey D. Finley

**Affiliations:** 1 Mork Family Department of Chemical Engineering and Materials Science, University of Southern California, Los Angeles, CA USA; 2 Departments of Pediatrics and Stem Cell Biology and Regenerative Medicine, Keck School of Medicine, University of Southern California, Los Angeles, CA USA; 3 Department of Biomedical Engineering, University of Southern California, Los Angeles, CA USA; 4 Department of Biological Sciences, University of Southern California, Los Angeles, CA USA

**Keywords:** Intracellular signaling, Feedback control, Ensemble modeling, Beta cell biology

## Abstract

**Introduction:**

The expansion of insulin-producing beta cells during pregnancy is critical to maintain glucose homeostasis in the face of increasing insulin resistance. Prolactin receptor (PRLR) signaling is one of the primary mediators of beta cell expansion during pregnancy, and loss of PRLR signaling results in reduced beta cell mass and gestational diabetes. Harnessing the proliferative potential of prolactin signaling to expand beta cell mass outside of the context of pregnancy requires quantitative understanding of the signaling at the molecular level.

**Methods:**

A mechanistic computational model was constructed to describe prolactin-mediated JAK-STAT signaling in pancreatic beta cells. The effect of different regulatory modules was explored through ensemble modeling. A Bayesian approach for likelihood estimation was used to fit the model to experimental data from the literature.

**Results:**

Including receptor upregulation, with either inhibition by SOCS proteins, receptor internalization, or both, allowed the model to match experimental results for INS-1 cells treated with prolactin. The model predicts that faster dimerization and nuclear import rates of STAT5B compared to STAT5A can explain the higher STAT5B nuclear translocation. The model was used to predict the dose response of STAT5B translocation in rat primary beta cells treated with prolactin and reveal possible strategies to modulate STAT5 signaling.

**Conclusions:**

JAK-STAT signaling must be tightly controlled to obtain the biphasic response in STAT5 activation seen experimentally. Receptor up-regulation, combined with SOCS inhibition, receptor internalization, or both is required to match experimental data. Modulating reactions upstream in the signaling can enhance STAT5 activation to increase beta cell survival.

**Electronic supplementary material:**

The online version of this article (10.1007/s12195-020-00647-8) contains supplementary material, which is available to authorized users.

## Introduction

Metabolic diseases impair the body’s ability to properly convert nutrients into energy. Diabetes is a particularly harmful metabolic disease that affects over 30 million people in the United States alone.^33^ While multiple factors contribute to the pathogenesis of diabetes, a deficit of functional insulin-secreting beta cells underlies all forms of diabetes. In cases of Type 1 diabetes, an autoimmune attack destroys the majority of beta cells, thus leaving patients unable to produce insulin, the key hormone that regulates the transport of glucose from the blood to the cells where it is used to produce energy. Patients with Type 2 or gestational diabetes can produce some insulin, but not enough to properly regulate blood glucose levels in the context of insulin resistance. Recent advances in the study of pancreatic beta cells have shed light on the body’s ability to adapt and expand in response to changes in metabolic demand.^36^ For example, in cases of high insulin resistance, such as pregnancy or obesity, the body maintains glucose homeostasis by increasing beta cell mass in the pancreas. In fact, studies have shown that over the approximately 20-day time course of pregnancy in mice, pancreatic beta cells both replicate and grow in size, resulting in an increased beta cell mass.^36^ The ability to induce beta cell expansion could be a powerful step to increase the number of functioning beta cells in diabetes patients.

Beta cell expansion is driven by signaling through the prolactin receptor^4^,^19^,^25^,^51^ (PRLR). Signaling by the lactogenic hormones prolactin and placental lactogen through PRLR stimulates the JAK-STAT signaling cascade.^35^ Specifically, Janus Kinase 2 (JAK2) is constitutively associated with the PRLR^7^,^17^,^38^ and once the JAK2 kinase is activated, it recruits and phosphorylates Signal Transducer and Activator of Transcription 5 (STAT5). STAT5 regulates the expression of several target genes in the nucleus, including genes related to the cell cycle^20^,^45^ and survival.^21^,^26^,^50^ Although initial discoveries were made in rodent models, human prolactin has been shown to increase beta cell survival as well.^50^

In this work, we investigate the mechanisms by which the pregnancy-related hormone prolactin (PRL) drives JAK-STAT signaling in pancreatic beta cells using a mathematical model of the signaling pathway. We focus our model on JAK2-STAT5 signaling that promotes beta cell survival mediated by the protein Bcl-xL. Experimental studies performed with the beta cell line INS-1, as well as primary cells from rodents and humans, demonstrate that signaling through JAK2-STAT5 promotes cell survival via Bcl-xL.^21^,^26^ For example, Fujinaka *et al*. demonstrated that Bcl-xL up-regulation induced by JAK2-STAT5 signaling promotes beta cell survival. They demonstrated that in both INS-1 cells and primary beta cells and showed that siRNA knockout of Bcl-xL inhibits lactogen-mediated protection from cell death. In addition, Silva *et al.*^41^ show that nuclear localization of STAT5 promotes Bcl-xL gene expression: they found direct binding of STAT5 to the Bcl-xL promoter. Since beta cell mass depends on both cell apoptosis and survival and Bcl-xL is required to mediate pro-survival effects in INS-1 cells and primary cells, there is a relationship between Bcl-xL and beta cell mass.

Mathematical models have been used to elucidate the balance between replication and apoptosis in beta cells,^30^ but no molecular-detailed computational model exists for the adaptive expansion of beta cells in response to pregnancy. Here, we use a systems biology approach to quantitatively analyze the beta cell response to hormone stimulation. In particular, we use mathematical modeling to explore the effects of various regulatory mechanisms that control signaling. Experimental data shows that when insulin-secreting cells of the INS-1 cell line are treated with a constant concentration of PRL *in vitro*, the amount of phosphorylated STAT5 (pSTAT5) has multiple peaks within six hours of stimulation.^10^,^11^ The presence of these peaks is influenced by Suppressors of Cytokine Signaling (SOCS) genes, which are transcribed in response to STAT signaling and exert negative feedback on the system. Modeling the cytokine IFN-γ in liver cells, Yamada *et al.* found that the presence of a nuclear phosphatase, in addition to SOCS negative feedback, are sufficient to cause a decrease in phosphorylated STAT after the initial peak, leading to multiple peaks in phosphorylated STAT dimer in the nucleus.^52^ The role of SOCS protein in inhibiting JAK-STAT signaling was further elucidated by Singh *et al.* through joint modeling of JAK-STAT and MAPK pathways in hepatocytes in response to IL-6.^42^ Particular to our system of study, JAK-STAT signaling through the prolactin receptor (PRLR) has been shown to include positive feedback as nuclear STAT5 promotes transcription of PRLR mRNA.^22^,^28^,^34^,^36^

We hypothesize that positive feedback could play a role in explaining the initial peak, subsequent decline, then prolonged activation of STAT5 activity in INS-1 cells discovered by Brelje *et al*. Although these regulatory mechanisms significantly influence beta cell signaling, no model to our knowledge explores the interplay between SOCS feedback and positive regulation of PRL signaling. Therefore, we built upon prior work in the field to create a computational model of signaling that promotes adaptive expansion of beta cell mass in response to pregnancy driven by JAK-STAT signaling in pancreatic beta cells through PRLR. Our work is distinct from previous research because we focus on a different cell type and calibrate our model using experimental data. Since the kinetics of the signaling pathways and the importance of different regulatory mechanisms are cell type-dependent, it is important to fit models to data from the cell type of interest. We fit our model directly to experimental data for STAT5 phosphorylation and localization in the INS-1 cell line. Additionally, we explored up-regulation of the prolactin receptor due to transcriptional activity of STAT5, a control mechanism that is particularly relevant to pancreatic beta cells and is shown in the experimental data from Brelje *et al*.^10^,^11^ This regulatory mechanism has not been explored in any previous computational models.

We applied the model to investigate the influence of these regulation mechanisms, individually and in combination, and found that model structures that include both positive and negative regulation produce multiple peaks in STAT5 phosphorylation within a tight range of parameter values. By fitting to experimental data using a Bayesian approach for likelihood estimation of parameter values, we show that the model can simultaneously predict STAT5 phosphorylation and nuclear translocation. The model predicts a faster dimerization and nuclear import rate for STAT5B dimers than STAT5A, which can explain their different activation profiles observed experimentally. Our experimentally-derived mathematical model provides a framework to quantitatively understand lactogenic signaling that mediates the adaptive expansion of beta cell mass during pregnancy.

## Results

### Mechanistic Model of JAK-STAT Signaling in Beta Cells

A mechanistic model of JAK2-STAT5 signaling through the prolactin receptor was constructed based on known reactions from the literature. The model builds on the prior work of Yamada *et al.* 2003 modeling control mechanisms in JAK-STAT signal transduction^52^ and Finley, *et al.* 2011, which analyzed IL-12 mediated JAK-STAT signaling in T cells.^18^

**Figure 1:**
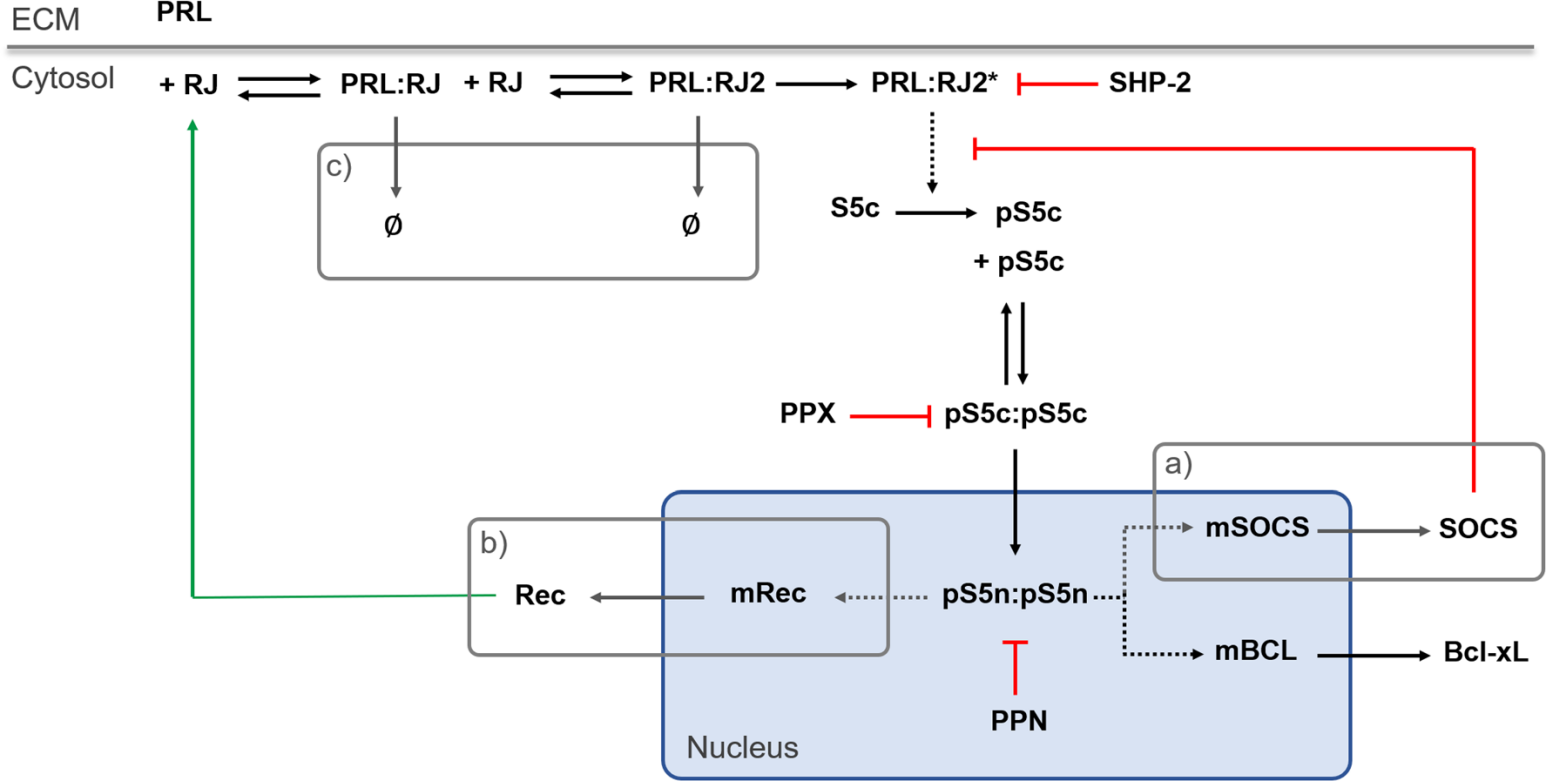
Model schematic of JAK-STAT signaling in pancreatic beta cells. PRL binds to the PRLR:JAK2 complex (RJ), which induces receptor dimerization and activation by JAK2 kinase activity. The activated receptor PRL:RJ2* phosphorylates STAT5, which dimerizes and transports into the nucleus, where it promotes transcription of target genes. Phosphatases attenuate the signaling at the membrane (SHP-2), in the cytosol (PPX), and in the nucleus (PPN). Signaling modules for ensemble modeling include (a) STAT5-induced SOCS negative feedback, (b) STAT5-induced receptor up-regulation, and (c) ligand-induced receptor internalization. Green indicates positive feedback; red indicates inhibition of signaling. *ECM*, extracellular matrix.

The mechanistic model includes a core network representing the canonical JAK-STAT signaling cascade, which includes 31 reactions and 24 molecular species (Fig. 1). Three regulatory modules are included or excluded from the network in order to consider their effect on STAT5 activation. These include (a) SOCS exerting negative feedback on STAT5 phosphorylation, (b) receptor up-regulation due to transcriptional action of phosphorylated STAT5, and (c) internalization of the prolactin receptor induced by ligand binding. Including each regulatory module individually and in all possible combinations leads to eight model structures to explore. The full signaling network with all three regulatory modules included has 47 reactions and 32 molecular species. A full list of reactions is included in the supplementary material File S1.

### Effect of Different Regulatory Modules on Qualitative Shape of pSTAT5 Activation

**Figure 2:**
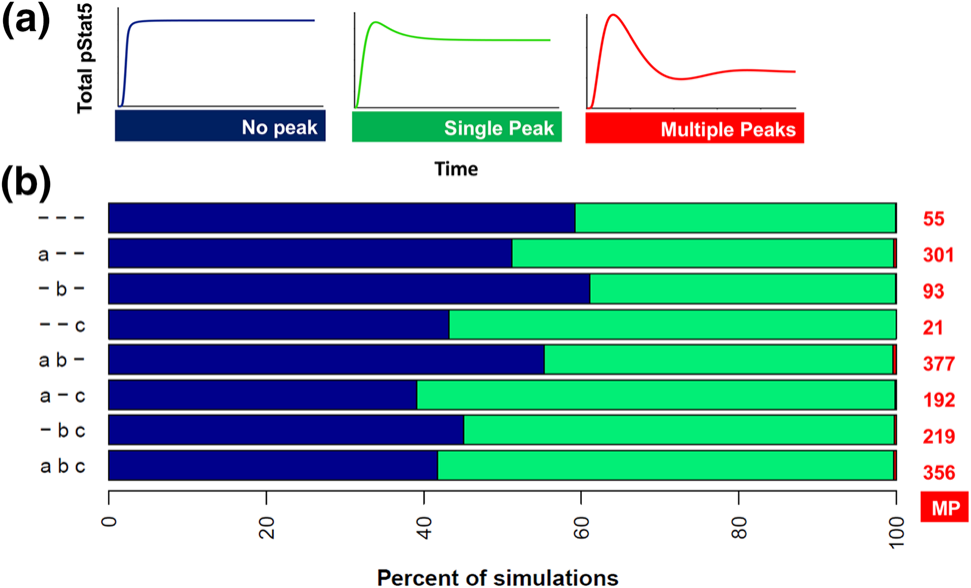
Ensemble Modeling Predicts the Number of Peaks in STAT5 Phosphorylation. (a) Simulated time courses were classified into three shapes based on the number of peaks in STAT5 phosphorylation over 6 hours of PRL stimulation. (b) Bar chart shows the percentage of Monte Carlo simulations from each model structure that were classified into each shape shown in panel A. Row labels correspond to the inclusion or exclusion of regulatory modules shown in Fig. 1. The data labels in red show the number of simulations that were classified as “Multiple Peaks” for each structure. *n* = 100,000 simulations per structure (800,000 total). *MP* Multiple Peaks.

We defined eight model structures based on inclusion or exclusion of the three regulatory modules from Fig. 1 and ran Monte Carlo simulations for each structure to explore model predictions across a wide area of the parameter space. Here, we varied all parameter values (i.e., the kinetic reaction rates) and non-zero initial conditions (see “Methods”). This enables us to efficiently explore the parameter space and characterize the simulated dynamics. Each simulation was classified as “No peak”, “Single Peak”, or “Multiple Peaks” based on the predicted time course of STAT5 phosphorylation (Fig. 2a). Within the model structures with only one regulatory module included, the structure that included SOCS feedback was most likely to show multiple peaks in STAT5 phosphorylation (Fig. 2b). The structure that included receptor internalization was most likely to produce a single peak in STAT5 phosphorylation. Overall, the likelihood of randomly sampled parameter sets producing a time course of STAT5 phosphorylation with multiple peaks was very low for all model structures. Of the 8 × 10^5^ simulations we performed, only 1614 (0.2% of simulations) exhibited multiple peaks. This indicates that tight control of the reaction rates is necessary to achieve the right balance of activation and attenuation.

From the Monte Carlo simulations, model predictions that had multiple peaks in STAT5 phosphorylation showed wide variation in the magnitude and time course of phosphorylation. Therefore, we set out to define a more detailed classification to understand which model structures could give rise to STAT5 dynamics matching those observed in INS-1 cells, which show a defined profile for phosphorylated STAT5. Specifically, Brelje and colleagues showed that STAT5 reaches an initial peak at approximately 30 min following the initial stimulation, followed by attenuation between 1 and 3 h, which reduces phosphorylation to below 70% of its initial peak. A second increase is observed after three hours, where phosphorylated STAT5 reaches or exceeds the initial level of phosphorylation.^10^,^11^ We first filtered the Monte Carlo simulations, retaining those that resulted in an appreciable level of STAT5 phosphorylation (at least 1% of the initial STAT5 becomes phosphorylated), assuming a minimum amount of phosphorylation is required to promote downstream signaling and cell response. We then defined eight qualitative shapes of STAT5 activation based on the number of peaks and the time at which the peaks occur. The decision tree used to classify predicted time courses is shown in Fig. S1.

**Figure 3:**
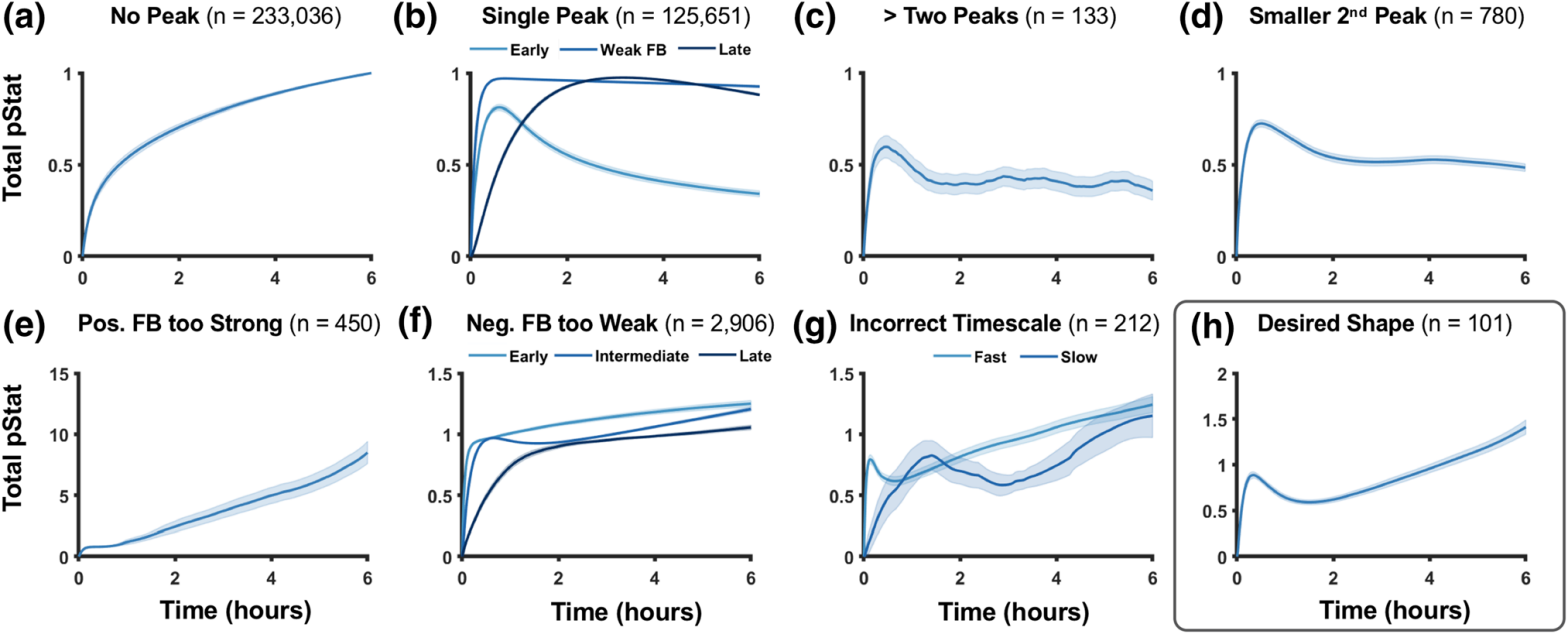
Classification of simulations into qualitative shapes. Simulated time course of STAT5 phosphorylation for each shape shows the mean (solid line) and 95% confidence interval (shaded area) of all Monte Carlo simulations (800,000 total) classified into that shape. All shapes are mutually exclusive, that is, all simulations were uniquely assigned to one shape (see Fig. S1 for decision tree). Simulations that did not reach a threshold level of 1% of STAT5 phosphorylated were labeled as “weak activation” and filtered out, *n* = 436,731.

This classification enabled a detailed characterization of the dynamics of phosphorylated STAT5. A large number of simulations had no peak in STAT5 phosphorylation (Fig. 3a) or attenuation of the initial STAT5 activation leading to a single peak in pSTAT5 (Fig. 3b). A select few parameter sets (0.09%) produced multiple peaks in STAT5 phosphorylation that did not match the qualitative shape of experimental data, such as having more than one oscillation within 6 h (Fig. 3c) or showing a smaller second peak characteristic of damped oscillation (Fig. 3d). This damped oscillation profile has been shown in prior modeling of JAK-STAT signaling^42^,^52^ but does not explain the activation profile observed in INS-1 cells treated with prolactin. Positive feedback can lead to unstable systems, and some simulations (0.05%) had an early peak in STAT5 phosphorylation followed by a large increase in phosphorylation due to strong positive feedback (Fig. 3e). Over 2000 (0.36%) simulations had an initial peak followed by minimal attenuation before reactivation (Fig. 3f). These simulations are grouped into early, intermediate, and late simulations to preserve the qualitative shape when pooling simulations together. Another small group of simulations (0.03%) had multiple peaks in pSTAT5 but did not match the time course of the experimental data, either because the reactivation was too fast (< 3 h) or the initial peak was too slow (> 1 h) (Fig. 3g).

**Figure 4:**
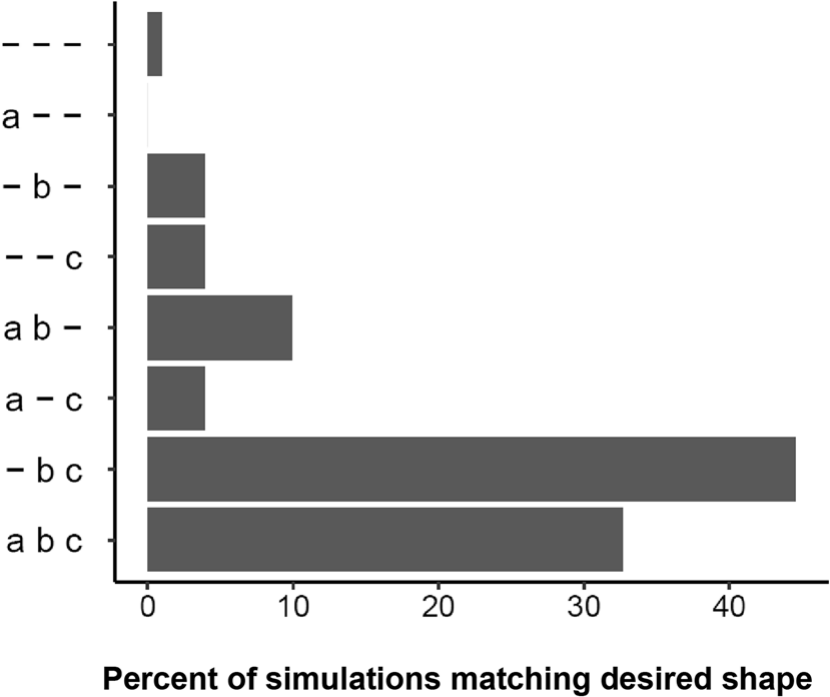
Breakdown of simulations matching desired shape by structure. The *y*-axis shows the eight model structures defined by the inclusion or exclusion of the regulatory modules. Horizontal bars show the percentage contribution of each model structure to the 101 simulations that matched the desired shape shown in Fig. 3h.

Finally, a small number of simulations (101) matched the qualitative shape of the experimental data (Fig. 3h). Simulations classified as having this desired shape comprise just over 0.01% of the 800,000 total simulations, pointing to the necessity of tightly controlled balance of positive feedback and negative feedback, both in terms of the strength and timescale of feedback. The eight distinct model structures contributed differently to the fraction of simulations that match the desired shape (Fig. 4). Although SOCS inhibition was sufficient to cause multiple peaks in STAT5 phosphorylation (Fig. 2), SOCS inhibition alone was not sufficient to cause an early peak followed by prolonged activation (Fig. 4, row 2). Model structures that included receptor up-regulation, combined with either SOCS inhibition, receptor internalization, or both had the highest likelihood of matching the desired qualitative shape (Fig. 4, rows 5, 7, and 8). We found that the likelihood of these three model structures to match the qualitative shape of STAT5 activation was similar even with noise in parameter values (Fig. S2).

### Effect of Parameter Values on Time Course of STAT5 Activation

**Figure 5:**
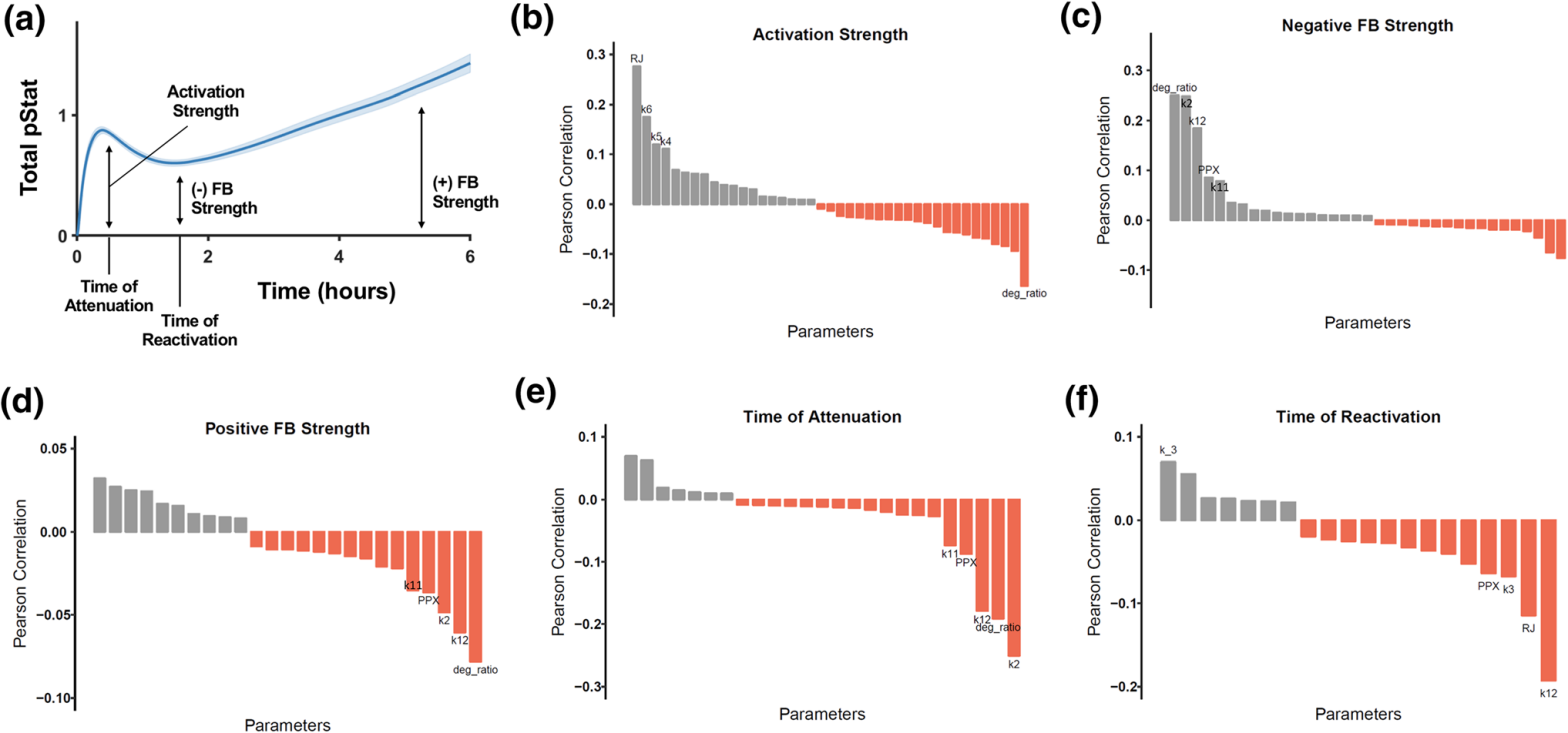
Parameters Correlated with STAT5 phosphorylation. Pearson correlation between each kinetic parameter or initial value and five quantitative characteristics of the STAT5 phosphorylation time course. (a) Illustration of five characteristics. (b) Activation strength. (c) Negative feedback strength. (d) Positive feedback strength. (e) Time of attenuation. (f) Time of reactivation. Only parameters with statistically significant (*p* < 0 .05) correlations are shown in the waterfall plots. The five parameters most highly correlated with each characteristic are labeled. ***RJ*** initial value of PRLR:JAK2 complex, ***k6*** phosphorylation rate of STAT5, ***k5*** activation rate of JAK2, ***k4*** dimerization rate of PRLR:JAK2 complexes, ***deg_ratio*** ratio of degradation rate of ligand-bound receptor complexes to unbound complexes, ***k2*** ligand binding *on* rate, ***k12*** rate of dephosphorylation of pSTAT5 by cytoplasmic phosphatase, ***PPX*** initial value of cytoplasmic phosphatase, ***k11*** binding rate of cytoplasmic phosphatase to pSTAT5, ***k_3*** receptor complex dimerization *off* rate, ***k3*** receptor complex dimerization *on* rate. The full list of correlated parameters and their Pearson correlation values are given in Supplemental File S1.

Kinetic parameter values affect the strength of STAT5 activation, strength of feedback, and timescale of feedback. Several parameters from Monte Carlo simulations were strongly correlated with characteristics of the predicted time course of phosphorylated STAT5 (Fig. 5a). These characteristics include the activation strength, the strengths of the negative and positive feedback, and the times of attenuation and reactivation (see “Methods” section for more detail). The Pearson correlation coefficients for each statistically significant association are shown in Figs. 5b to 5f. For ease of viewing, we labeled the five parameters in each panel that had the highest absolute value of correlation coefficient and include the correlation and *p*-value for all parameters in Supplemental File S1. The ratio of the ligand-bound receptor degradation rate to the unbound receptor degradation rate (*deg_ratio*) was highly correlated with four of the five defined characteristics of the pSTAT5 time course. As expected, higher values of *deg_ratio* decreased the activation strength (Fig. 5b), increased the strength of negative feedback (Fig. 5c), and decreased the strength of positive feedback (Fig. 5d). Increased values of *deg_ratio* also led to a shorter timescale of attenuation (Fig. 5e) because the active receptor complex had a shorter half-life in the cell and therefore less time to phosphorylate STAT5. The parameter *k2*, the ligand-receptor binding rate, had a similar effect as *deg_ratio* on the strength of feedback and time scale of attenuation (Figs. 5c to 5e). and timescale of feedback (Figs. 5c to 5f). However, it was positively correlated with the strength of activation (see Supplemental File S1). A faster rate of ligand binding leads to a stronger activation but stronger negative feedback due to increased internalization of ligand-bound receptors.

Increased values of the initial concentration of the receptor:JAK2 complex (*RJ*) increased the activation strength (Fig. 5b) and shortened the time of reactivation (Fig. 5f). With more receptor complexes at the surface, a larger fraction of STAT5 can be phosphorylated initially and upon reactivation after attenuation of the initial signal. Predictably, parameters that govern the rate of interactions critical to STAT5 activation (*k4*, *k5*, and *k6*, corresponding to the rate at which the ligand-bound receptor complex is activated, binds STAT5, and phosphorylates STAT5, respectively) were positively correlated with the activation strength (Fig. 5b). Additionally, increases in *k12*, the rate at which cytosolic phosphatase dephosphorylates STAT5, led to stronger negative feedback (Fig. 5c), weaker positive feedback (Fig. 5d), and a faster timescale of attenuation (Fig. 5e). Overall, this analysis provides mechanistic insight into how specific biochemical reactions influence key features of STAT5 dynamics. Such results can guide experimental studies to modulate the signaling network to enhance STAT5 response.

### Model Calibration to STAT5 Dynamics in INS-1 Cells

The results presented thus far provide a detailed analysis of the model features that give rise to STAT5 dynamics that qualitatively agree with experimental data. We next aimed to produce a predictive model that quantitatively matches the data by calibrating the computational model to the experimental data. Specifically, we fit the model to measurements for the time course of phosphorylation of JAK2, STAT5A and STAT5B,^11^ translocation of STAT5A and STAT5B from the cytoplasm to the nucleus,^11^ and the fold change in protein level of the pro-survival response protein Bcl-xL.^21^

We chose to fit the three model structures that produced a reasonable number of simulations (> 5%) matching the qualitative shape of STAT5 activation from ensemble modeling. This included the full model with all regulatory mechanisms (Fig. 4, row 8) as well as the model structure that did not include receptor internalization (Fig. 4, row 5) and that did not include SOCS negative regulation (Fig. 4, row 7). The model structure that included all three regulatory modules had the lowest minimum Sum of Squared Errors (SSE) and median SSE (Table 1). In addition to using the SSE to evaluate the model fits, we also use the Akaike Information Criterion (AIC), which allows for comparison of model structures with different number of fitted parameters, penalizing structures that have more parameters.^37^,^44^ A lower value of AIC indicates a better fit. The model structure without SOCS negative feedback had the lowest AIC. This structure fit the data similarly well as the full model (Fig. S4) and has a lower number of fitted parameters, leading to a lower AIC. Our modeling predicts that SOCS negative regulation is not necessary for early activation, attenuation, and reactivation of STAT5 in pancreatic beta cells treated with prolactin. Other sources of negative regulation such as phosphatase action and internalization of ligand-bound receptors, combined with positive regulation due to STAT5-induced receptor upregulation, can drive the experimentally observed activation profile.

**Table 1:** Comparison of model structures.

	*ab-*	*-bc*	abc*
Range of SSE	0.595–0.751	0.585–0.687	0.424–0.643
Median SSE	0.662	0.639	0.602
AIC (ΔAIC)^†^	− 79.85 (9.25)	− 89.11 (0)	− 81.25 (7.85)

*Columns represent the model structures defined by inclusion or exclusion of regulatory modules a, b, c from Fig. 1. A dash represents exclusion of a given regulatory module

^†^Δ AIC is the AIC for each model structure minus the AIC for the structure with lowest AIC, which is structure **-***bc*

**Figure 6:**
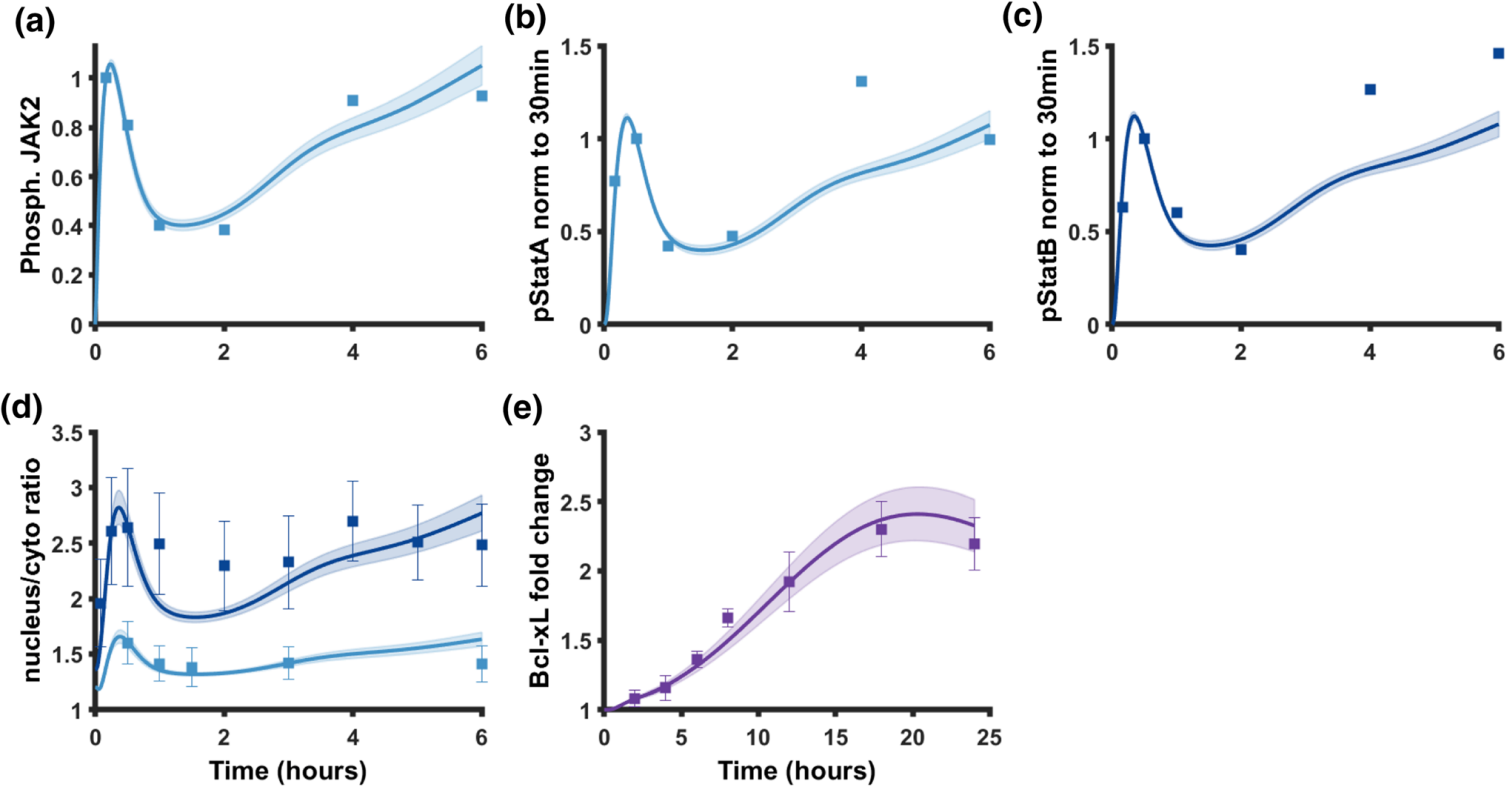
Model calibration. Model predictions for (a) Phosphorylated JAK2, normalized to the 10 min time point, (b) Phosphorylated STAT5A, normalized to the 30 min time point, (c) Phosphorylated STAT5B, normalized to the 30-min time point, (d) Ratio of nuclear to cytosolic STAT5A and STAT5B, and (d) Fold change of Bcl-xL. Lines show mean value of model predictions with shading indicating the standard deviation across the 1000 parameter sets from the posterior distribution. Squares show experimental data points from Brelje *et al.* for panels A, B, C, and D or from Fujinaka *et al.* for panel E. Error bars are included for experimental data points that had error bars shown in the previously published work. All experimental data are for INS-1 cells treated with PRL at 200 ng/mL. Thirty-three parameters were fit simultaneously to the six data sets using a Bayesian likelihood estimation approach. *Dark blue,* STAT5A; *light blue,* STAT5B in (d).

We focus our analysis on the model that included all three regulatory modules, as that model structure produced the lowest SSE and allowed us to probe each different regulatory mechanism. Model predictions for this full model are shown in Fig. 6, illustrating that this structure effectively captured the phosphorylation dynamics of JAK2 (Fig. 6a), STAT5A (Fig. 6b), and STAT5B (Fig. 6c) as well as the nuclear import (Fig. 6d) of both STAT5A and STAT5B on the six-hour timescale. The dynamics of STAT5 phosphorylation and nuclear import share a similar qualitative shape because phosphorylation is necessary for shuttling of STAT5 to the nucleus.

Interestingly, although STAT5A and STAT5B show a similar time course of phosphorylation, they differ in the amount that is translocated into the nucleus.^10^,^11^ The model accounts for separate STAT5A and STAT5B species and allows for homo- and hetero-dimerization with separate rate constants for dimerization, import of phosphorylated dimers into the nucleus, and export of dephosphorylated STATs from the nucleus. The fitted model predicts that STAT5B homodimers form faster than STAT5A homodimers, with a ratio of 4.24 ± 0.03 as compared to the STAT5A dimerization rate. The model also predicts the nuclear import rate to be faster for STAT5B homodimers than STAT5A homodimers, with a ratio of 4.94 ± 0.03 as compared to the STAT5A nuclear import rate. The faster dimerization rate and nuclear import rate predicted by the model provide a potential hypothesis for greater STAT5B nuclear localization as compared to STAT5A, which has been observed experimentally.

In addition to predicting the upstream dynamics, the model also predicts the fold change of Bcl-xL, a response protein that is induced by pSTAT5 activity in the nucleus (Fig. 6e). The model predicts that the fold change of the pro-survival protein Bcl-xL increases through 18 h of stimulation with prolactin before decreasing after 18 h, matching experimental observations from Fujinaka *et al.*^21^ that capture how a single oscillation in STAT5 activation on the six hour timescale can lead to a smooth increase in the concentration of a response protein on a longer timescale (Fig. 6e). Taken as a whole, the fitting results suggest that multiple feedback mechanisms could explain the observed time courses in STAT5 phosphorylation, nuclear translocation, and protein response. However, receptor upregulation is required, and it must be combined with at least one of the other regulatory mechanisms (SOCS negative feedback or receptor internalization). The calibrated model containing all three regulatory modules produces the best fit to the data and generates consistent parameter estimates (Fig. S6).

### Dose Response Predictions for Beta Cells Treated with Prolactin

**Figure 7:**
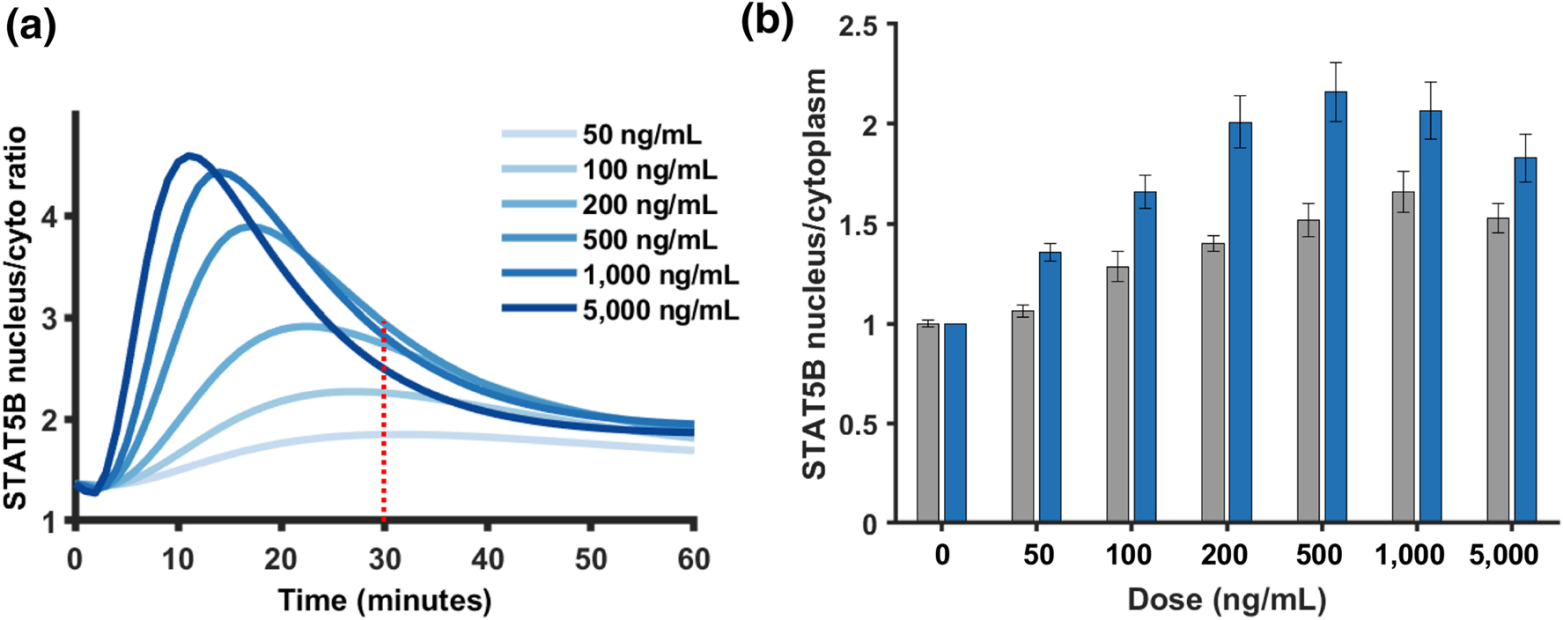
Dose Response predictions. (a) Model predicted time course of STAT5B import into the nucleus under various concentrations of PRL ligand, simulated for 60 minutes. The red dotted line emphasizes the values at the 30 minute time point, which is plotted in the bar chart in panel B. **(b)** Model predicted dose response data for 30-min timepoint (blue) compared to experimental data from Brelje *et al.* treating rat primary beta cells with PRL (grey). Values are normalized to the amount of STAT5B in the nucleus with no PRL stimulation (0 ng/mL dose). Error bars for model predictions show standard deviation of predictions across the 1,000 posterior parameter sets.

We next aimed to use the parameterized model to make new predictions for STAT5 signaling through the prolactin receptor. We tested six concentrations of prolactin used by Brelje *et al.*^11^to treat rat primary beta cells *in vitro* and found that higher concentrations of prolactin lead to a greater magnitude of STAT5B translocation to the nucleus and an earlier peak in STAT5B translocation (Fig. 7a). We quantified the amount of STAT5B translocation at the 30 min time point in order to compare to experimental measurements using immunohistochemistry from Brelje *et al.* We found that the model predictions match the qualitative shape of the experimentally determined dose response curve (Fig. 7b), showing a biphasic response, in which the STAT5B level increases with increasing stimulation before decreasing. However, there are differences between the model predictions and experimental data. Specifically, the model predicts an increase in STAT5B translocation at the 30-min timepoint (Fig. 7b, blue bars) with increasing hormone concentration, with the maximal response occurring at a dose of 500 ng/mL. In comparison, the peak response occurs at the 1000 ng/mL dose in the experimental data (Fig. 7b, grey bars). Given that the model produces the full time-course of STAT5 levels, we can investigate why there is this difference between model and experiments. The model predicts that the attenuation of the initial STAT5 activation occurs more rapidly for higher doses of PRL such that attenuation has already reduced STAT5 levels by the 30-min timepoint (Fig. 7a). This difference in the timing may be due to having calibrated the model using data from INS-1 cells treated with prolactin rather than rat primary beta cells. Multiple studies point to differences in enzyme catalytic rates in different biological settings.^13^,^54^

### Perturbing the Fitted Model

Next, we examined the influence of varying individual parameters and initial values on model predictions. We varied each parameter or initial value that was determined by ensemble modeling to have a large impact on one of the various aspects of STAT5 activation (large correlation values in Figs. 5b to 5f) individually within two orders of magnitude of the fitted values. Changing individual parameter and initial values altered both the strength of activation and the feedback dynamics, suggesting that the feedback system can be modulated.

**Figure 8:**
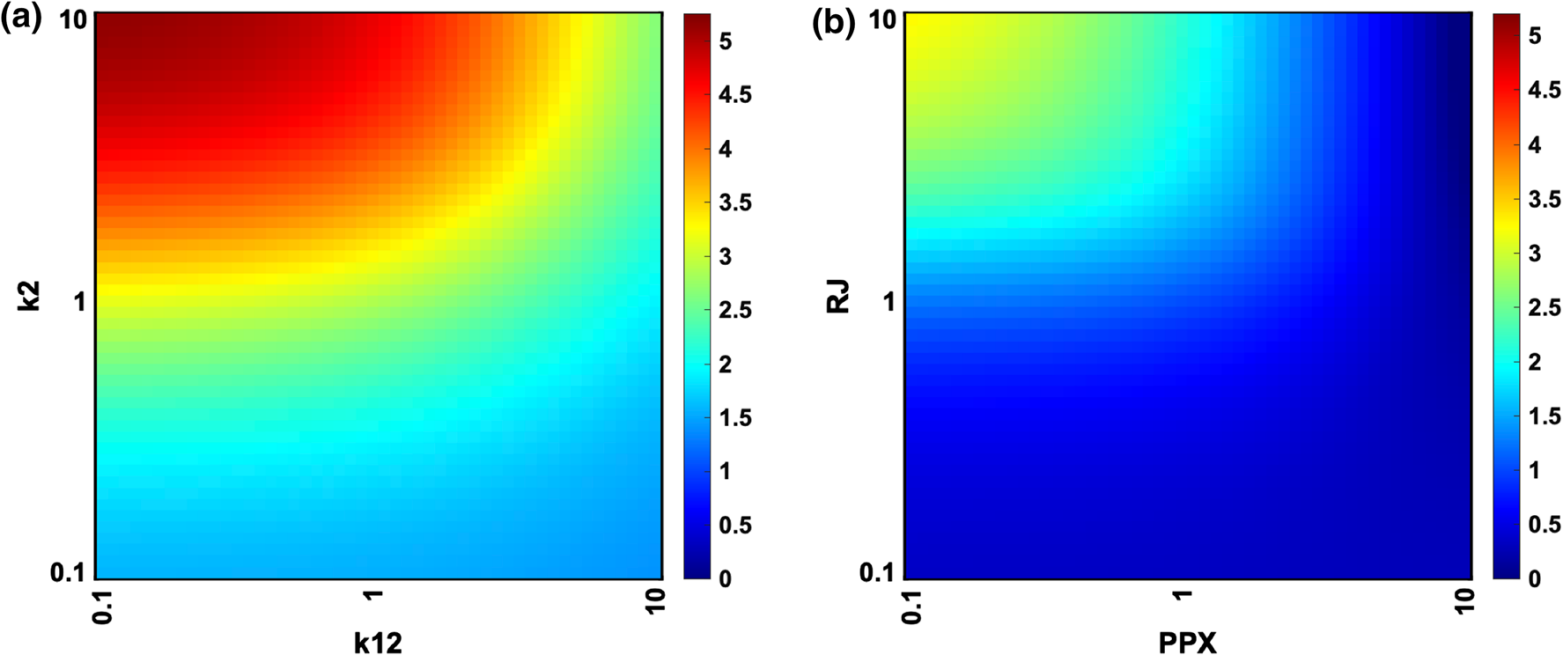
Model perturbations. (a) The effect of varying the initial ligand-binding rate *k2* and the cytosolic phosphatase dephosphorylation rate *k12* between 0.1-fold and 10-fold of the fitted parameter values. **(b)** Varying the initial values of the receptor-JAK2 complex *RJ* and the cytosolic phosphatase *PPX* between 0.1- and 10-fold of the fitted values. Coloring of the heat map indicates the initial peak in the STAT5B cytoplasm to nucleus ratio averaged across the 1000 posterior parameter sets.

We chose to investigate in detail two of the highest ranking influential kinetic parameters and initial values, based on their ability to strongly modulate multiple aspects of STAT5 activation. We predicted the time course of model species in response to changes in parameter values (Fig. S12) and quantified the initial peak in STAT5B nucleus to cytoplasm ratio, which represents the strength of activation of the system (Fig. 8). When varying the PRL ligand binding rate (*k2*) and the cytosolic phosphatase dephosphorylation rate (*k12*) two orders of magnitude, we found that the activation of STAT5 was more sensitive to changes in the ligand binding rate, as indicated by the increase in activation along the *y*-axis (Fig. 8a). The phosphatase did modulate activation, with higher values of *k12* leading to lower activation, but the effect is less pronounced than that of *k2*. A similar result was obtained when varying the initial value of the receptor complex (*RJ*) and the cytosolic phosphatase (*PPX*). The activation was increased greatly when the initial value *RJ* approached ten times its fitted value (Fig. 8b). Higher initial values of *PPX* decreased the strength of initial value, but again, this effect is less pronounced than modulating signaling at the receptor level. In this case, when the initial concentration of *RJ* is too low or that of *PPX* is too high, there is no distinct peak for the nuclear to cytoplasmic ratio of STAT5B (indicated by the value 0 in Fig. 8b). This can be explained, as low *RJ* would prevent the prolactin input signal from being transduced, and high *PPX* would strongly attenuate the signal.

Based on these simulation results, we conclude that targeting reactions upstream in the signaling network has a larger impact on the activation of STAT5, as compared to directly targeting regulatory reactions in the signaling cascade. Interestingly, changing these two sets of parameters produces nonlinear effects, as indicated by the curved isoclines in Fig. 8. Overall, the model is useful in predicting how altering kinetic parameters and species’ concentrations influences signaling dynamics that directly mediate pro-survival signaling.

## Discussion

Our mechanistic model of JAK-STAT signaling in pancreatic beta cells captures key dynamics of STAT5 activation via phosphorylation by the PRLR-JAK2 complex, followed by import of phosphorylated STAT5 dimers into the nucleus. The model differentiates between STAT5A and STAT5B and identifies the kinetic rate parameters that are able to explain experimentally observed differences in the amount of the STAT5A and STAT5B entering the nucleus under prolactin stimulation.^10^,^11^ Specifically, the model shows that the rates of dimerization and translocation can account for the experimental measurements. This mechanistic insight is relevant, as it has been hypothesized that this differential expression of STAT5A and STAT5B in the nuclear and cytosolic forms may be a form of tissue-specific regulation of JAK-STAT signaling,^2^ arising from their differential affinity for STAT5 target genes.^6^ The model simultaneously predicts experimental data for upstream activation of STAT5 and fold change of the response protein Bcl-xL to the same hormonal stimulus.

In addition, the model was used to predict the dose response of STAT5 nuclear import under different concentrations of prolactin. This demonstrates the predictive capability of the model since the simulated dose response curve qualitatively matched the experimentally observed dose response data, which was not used for parameter estimation. In addition, we establish that although the model was calibrated using data from INS-1 cells, it can reproduce observations obtained using rat primary pancreatic beta cells. This is a particularly important point since INS-1 cells, while used as a model of primary beta cells, exhibit quantitative differences in their metabolism^46^ and insulin secretion in response to glucose,^32^ as compared to healthy beta cells.

The model includes reactions known to drive JAK-STAT signaling in pancreatic beta cells. There are a multitude of feedback modules affecting the signal transduction pathway,^35^,^40^ and we chose to explicitly explore the role of different feedback modules on the activation of STAT5 through ensemble modeling. We hypothesized that positive feedback through STAT5-induced receptor up-regulation could explain the reactivation of STAT5 in INS-1 cells to a magnitude greater than the initial activation.^10^,^11^ Classifying Monte Carlo simulated time courses by their qualitative shapes revealed that model structures with both receptor up-regulation and an inhibitory module (whether that be SOCS feedback of receptor internalization) were most likely to show reactivation of STAT5 matching the shape of the experimental data. Quantifying the impact of different parameter values on the time course of STAT5 activation helped us define which parameters drive the dynamics. An increased ligand-bound receptor degradation rate, for example, decreased the strength of activation and timescale of feedback while increasing the negative feedback strength. We followed up on the most influential parameters from ensemble modeling by varying them within the fitted model. Our simulations predict that modulating signaling at the receptor level produces larger increases in STAT5 activation than altering the effect of an individual feedback mechanism (cytosolic phosphatase). This information is relevant for researchers aiming to enhance beta cell survival through activation of the JAK-STAT pathway. Ultimately, we found that multiple model structures could fit the data well (Table 1), but there were emergent properties that were consistent across model structures, such as a faster rate of STAT5B dimerization and nuclear import, as compared to STAT5A.

We acknowledge some limitations of our work. Although the model predictions reproduce key aspects of the activation profile of STAT5 in INS-1 cells treated with prolactin, the model does not match experimental data for the 4-hour time point of pSTAT5A and the 4 and 6-h time points of pSTAT5B (Figs. 6b, and 6c). Although error bars were not included in the literature-derived data,^11^ we expect that there is greater uncertainty in phosphorylation measurements at later time points, as indicated by different quantitative values for pSTAT5A and pSTAT5B when the authors repeated the experiment in INS-1 cells in future work.^10^,^11^ Additionally, the model predicts a larger decrease in STAT5B upon initial attenuation than experimental data implies and a larger upward trajectory between 4 and 6 h (Fig. 6d). These discrepancies between model predictions and experimental data are likely due to the fact that in our model, STAT5 cannot be shuttled into the nucleus unless phosphorylated first, so dynamics of nuclear import are tied to the phosphorylation dynamics. In a living cell, additional factors likely affect the nuclear import of STAT5 such as other signaling pathways and concentration of importin proteins, which were not accounted for in our computational model. Our model predicts JAK-STAT signaling within the cell. Further work is required to integrate signaling across many cells to understand how JAK-STAT signaling can drive changes in beta cell mass. Lastly, we only consider JAK-STAT signaling through the PRLR. Other pathways, such as PI3K and MAPK cascades, have shown to be important in beta cell signaling.^8^,^27^,^47^ Future work can build on our model of JAK-STAT signaling to encompass other signaling pathways as well, as has been done for the JAK-STAT and MAPK activation in response to IL6 in hepatocytes.^42^

Despite these limitations, our model motivates new experiments that can better elucidate the role of regulatory elements in JAK-STAT signaling. Previous work has demonstrated that using principles of optimal experimental design can reduce uncertainty in parameter estimation.^3^,^43^ Based on our findings, multiple possible inhibitory mechanisms could explain the observed time course of STAT5 phosphorylation. By designing a time course stimulus of PRL on INS-1 cells that aims to discriminate between these different model structures, one could experimentally test which mechanism is most likely to occur within the cell. This in-depth exploration of signal transduction would benefit pre-clinical researchers trying to design a therapy aimed at increasing beta cell mass in model organisms of diabetes.

Taken as a whole, our work points to the importance of regulatory modules in JAK-STAT signaling within pancreatic beta cells. Our model predicts that positive feedback combined with inhibition, be that through negative feedback or enhanced degradation rate, can drive a single oscillation in STAT5 phosphorylation within 6 h, followed by a second peak that is higher than the first. Based on the rarity of this behavior occurring within the wide parameter space sampled, we contend that the kinetic rate parameters within the cell must be well constrained to balance positive and negative feedback and achieve this behavior. In line with this hypothesis, the kinetic parameters predicted by our model when fitting to experimental data were tightly constrained (Figs. S5–S7).

Excitingly, the mechanistic insight as to the detailed effects of the regulatory modules provides quantitative information needed to identify strategies to increase beta cell survival. The ability to increase the beta cell mass *in vivo* could be a powerful new therapy for the treatment of diabetes.^14^ Hormonal stimulus seeks to recapitulate the islet adaptation to pregnancy^5^ and has already achieved beta cell proliferation in rodent models^9^ in both female and male rodents.^24^ Despite these advances, potential therapies have failed to realize the same gains in beta cell proliferation in humans,^8^,^12^,^27^,^47^ pointing to a need for better understanding of regulatory mechanisms through the PRLR-JAK-STAT pathway.^12^ Here, we provide evidence that feedback modules play a key role in regulation of JAK-STAT signaling within a computational model relevant to the pancreatic beta cell. We also show that modulating upstream parameters such as the ligand binding rate and the initial value of receptor complexes can increase PRL-mediated STAT5 activation. We acknowledge the dependence of our model predictions on the accuracy of the model structure, and therefore explored several potential structures through ensemble modeling. The inclusion and exclusion of different regulatory modules gives insight into their relative importance and helps us understand the important predicted behaviors that emerge across multiple model structures.

## Methods

### Model Construction

A mathematical model was constructed to describe the reaction kinetics of JAK2 and STAT5 signaling in pancreatic beta cells. The model is comprised of ordinary differential equations, which describe how the concentrations of the molecular species in the reaction network evolve over time. Our model builds on the reactions and kinetic parameters from the work of Yamada *et al.*, who modeled control mechanisms of the JAK-STAT pathway in response to interferon-γ (IFN- γ) signaling.^52^ The model was adapted to include 2:1 ligand to receptor stoichiometry, which has been shown for the binding of prolactin (PRL) to the prolactin receptor (PRLR).^7^,^17^ Literature evidence shows that in humans^16^ and rats,^15^ prolactin has cyclic dynamics and rhythmic secretion. The timescale of these dynamics is likely different in the *in vitro* setting; however, to account for a decrease in prolactin levels over the timescale considered here, we included prolactin degradation. In the absence of experimental data for the half-life of prolactin, we assumed it is similar to that of estrogen (5–6 h) in MCF-7 cells.^39^ The receptor is assumed to be pre-associated with JAK2 (represented by the species RJ) since JAK2 is constitutively associated with the prolactin receptor.^7^,^17^,^38^ Once two RJ complexes are bound to one PRL hormone, the complex becomes activated. The receptor complex RJ has degradation and synthesis rates corresponding to a half-life on the cell membrane of 45 min.^10^ Once the ligand is bound, the receptor has a higher degradation rate, which represents internalization of the ligated receptor to the endosome.^1^,^7^,^10^

The activated receptor complex binds to the cytosolic form of STAT5 reversibly, and once bound, releases a phosphorylated form of STAT5 due to the kinase activity of JAK2. The pSTAT5 molecules dimerize in the cytosol and are transported into the nucleus. Three phosphatases are included in the model, which serve to attenuate the signaling after initial ligand binding: SH2 domain-containing tyrosine phosphatase 2 (SHP-2) dephosphorylates the activated receptor-JAK complex, and phosphatases in the cytosol and nucleus (termed PPX and PPN, respectively) dephosphorylate STAT5 species.^52^ The phosphatase action is a form of negative feedback shown to be necessary for attenuation of STAT activation.^52^ pSTAT molecules are shuttled out of the nucleus when they are not dimerized with another molecule. The phosphorylated STAT5 dimer promotes transcription of several target genes once in the nucleus. Specifically, we include SOCS, the prolactin receptor, and the pro-survival protein Bcl-xL as STAT5 targets. It has been shown that SOCS proteins bind competitively to the receptor JAK complexes and also target the receptors for ubiquitination-based degradation.^7^,^53^ These mechanisms were incorporated in the model rather than the non-competitive binding used by Yamada *et al*.^52^

STAT5 dimers promote transcription of mRNA for the prolactin receptor. This has been shown *in vitro* in INS-1 cells^22^ and *in vivo* during pregnancy in mice. This positive feedback mechanism may play a role in the islet response to pregnancy^22^ and has not been explored computationally before. The phosphorylated STAT5 dimer in the nucleus also promotes transcription of cell-cycle genes such as cyclin D proteins^20^,^45^ and anti-apoptotic species such as Bcl-family proteins.^21^,^50^ We included a module for the STAT5-mediated transcription and translation of the response protein Bcl-xL. A full list of reactions is included in the supplementary File S1. MATLAB was used to carry out model simulations, and statistical analyses of the simulated results were performed using R statistical computing language.^49^ All of the code necessary to run the simulations and produce all figures is publicly available at: https://github.com/FinleyLabUSC/JAK-STAT-Regulation-CAMB.

### Ensemble Modeling

The three optional modules (Fig. 1) were included or excluded from the core model. The induction of SOCS in response to STAT5 activation and its subsequent negative feedback on JAK-STAT signaling was the first optional module. The positive regulation due to up-regulation of the PRL receptor in response to activated STAT5 was the second optional module. The third optional module was receptor internalization, as represented by an enhanced degradation rate for ligand-bound receptors. The three optional modules were included in different combinations to produce eight possible model structures.

For each model structure, 100,000 Monte Carlo simulations were performed by sampling all free parameters and initial values from a log-uniform distribution. The parameters and initial values were varied two orders of magnitude above and below the initial guess (taken from previous models and literature evidence—see Supplementary File S1 “Parameters” and “Initial Values” spreadsheets). The total amount of phosphorylated STAT5 was calculated by summing together all forms of pSTAT5 and multiplying by two if the molecule included a STAT dimer with both STAT molecules phosphorylated.

We analyzed the features of the pSTAT5 concentration over time. The definitions of the characteristics of the pSTAT activation illustrated in Fig. 5a are as follows:

$${\text{Activation strength}} = \frac{{\text{Maximum pSTAT}}}{{\text{Total pSTAT}}}$$

$${\text{Negative FB Strength}} = 1 - \frac{\text{Minimum pSTAT after first peak}}{\text{pSTAT at first peak}}$$

$${\text{Positive FB Strength}} = \frac{\text{Maximum pSTAT after first peak}}{\text{pSTAT at first peak}}$$

$${\text{Time of attenuation}} = {\text{Time }}\left( {{\text{hr}}.} \right)\,{\text{of first peak}}$$

$${\text{Time of reactivation}} = {\text{First time}} \left( {{\text{hr}}.} \right)\,{\text{in which pSTAT goes from decreasing to increasing}}$$

The number of peaks in total pSTAT5 was quantified using the Matlab *findpeaks* function, which returned the value of total pSTAT5 at local maxima as well as the time of the peak in hours. Thresholds for the *findpeaks* function were defined to have a minimum distance between peaks of 20 min and a minimum peak prominence of 0.1% to avoid identifying noise in the data as peaks (see MATLAB *findpeaks* documentation).

A detailed shape classification was performed based on the decision tree in Fig. S1 implemented through *if* statements in our MATLAB script. Parameter correlations were calculated in R using the *cor* function. The correlations shown in Fig. 5 are calculated using Monte Carlo simulations from the full model structure that included all three regulatory modules. Correlations with activation strength were calculated using all 100,000 simulations. Correlations with negative FB strength, positive FB strength, and time of attenuation could only be calculated for simulations that had a peak, *n* = 58,265. Correlations with the time of reactivation could only be calculated for simulations that had reactivation, *n* = 11,123.

### Model Calibration

#### Sensitivity Analysis

A total of 33 parameters were chosen to fit to the 37 experimental data points based on a global sensitivity analysis. We used the extended Fourier Analysis Sensitivity Test (eFAST) to determine which parameters significantly influence the model predictions.^31^ The eFAST method uses a variance decomposition method to determine the sensitivity of model outputs to model inputs. The first-order sensitivity *S*_*i*_ quantifies the fraction of variance in model output that is explained by the input variance in the parameter *i*.

$$S_{i} = \frac{{\sigma_{i}^{2} }}{{\sigma_{\text{total}}^{2} }}$$

We calculated the first-order sensitivity of each kinetic parameter and non-zero initial value, with the output being all species’ concentrations predicted by our model. We also estimated the total-order sensitivity *S*_Ti_ for each kinetic parameter and initial value. *S*_Ti_ is calculated as one minus the summed sensitivity index of complementary parameters *S*_Ci_ which is defined as all parameters except parameter *i*.

$$S_{\text{Ti}} = 1 - S_{\text{Ci}}$$

In order to determine which parameters to fit to experimental data, we compared the total-order sensitivity index for all kinetic parameters and initial values on the predicted model outputs: phosphorylated STAT5A (pSTATA), phosphorylated STAT5B (pSTATB), nuclear to cytoplasm ratio of STAT5A (STAT5A_n_/STAT5A_c_), and the nuclear to cytoplasm ratio of STAT5B (STAT5B_n_/STAT5B_c_). Although we calculated *S*_*Ti*_ for each parameter on all model outputs, we chose to focus on the effect of each parameter on those four model predictions because they are used in the objective function in model calibration (see below).

We took the mean *S*_Ti_ for each parameter or initial value over each of the four model outputs listed above at each timepoint for which we had experimental data from the literature. These sensitivity indices are included in the Supplementary File S1 on the sheet “Sensitivity Analysis.” The parameters and initial values that had a mean *S*_Ti_ greater than that of the dummy variable, a factitious input which has no effect on model structure, were chosen as parameters to be fitted. In addition, the parameter *k30a*, which is the maximal rate of transcription of the PRLR receptor in response to STAT5 binding, was added to the parameter list because no kinetic parameter affecting the positive feedback module emerged from sensitivity analysis. In order to deconvolute the fact that the dimerization and shuttling rates of the different forms of STAT5 would likely be correlated, we defined the following multiplicative factors:

$$mult8B = \frac{k8B}{k8A}, \quad mult8AB = \frac{k8AB}{k8A}$$

$$mult14B = \frac{k14B}{k14A},\quad mult14AB = \frac{k14AB}{k14A}$$

$$mult17B = \frac{k17B}{k17A}$$

The parameters *k8A* and *k8B* describe the rate of homodimerization of STAT5A and STAT5B respectively while *k8AB* represents the rate of heterodimerization. The parameters *k14A, k14B,* and *k14AB* represent the rate of nuclear import of dimerized STAT5A dimers, STAT5B dimers, and heterodimers respectively. The parameters *k17A* and *k17B* represent the nuclear export rate of unphosphorylated STAT5A and STAT5B respectively.

#### Parameter Estimation

Parameter fitting was performed by fitting the model simultaneously to all of the experimental data used for likelihood estimation. The amount of phosphorylated JAK2, phosphorylated STAT5A, and phosphorylated STAT5B at the 10 min, 30 min, 1 h, 2 h, 4 h, and 6 h timepoints were quantified using Plot Digitizer (Java) from Brelje *et al.* Fig. 7.^11^ The nucleus to cytoplasm ratio of STAT5A and STAT5B at the 30 min, 1 h 1.5 h, 3 h, and 6 h. timepoints and the 5 min, 15 min, 30 min, 1 h, 2 h, 3 h, 4 h, 5 h, and 6 h. timepoints respectively were quantified using Plot Digitizer from Brelje *et al.* Fig. 6 results for INS-1 cells.^11^ The fold change of the anti-apoptotic protein Bcl-xL in response for the timepoints 2, 4, 6, 8, 12, 18, and 24 h were quantified using Plot Digitizer on Fujinaka *et al.* Fig. 7e.^21^ All experimental data from both papers was for INS-1 cells treated with 200 ng/mL of PRL.

A total of 50 independent fits were performed for each model structure using a Bayesian approach for likelihood estimation.^23^,^48^

Our group recently used this approach to calibrate a model of Natural Killer cell signaling,^29^ and we implemented the same algorithm in the current study. We assume the parameters are random variables and estimate the distributions of their values using a Bayesian. Thus, we maximized the posterior density $f\left( {\theta \left| y \right.} \right)$ of the parameters, *θ*, given the available experimental data, *y*, using the Metropolis-Hastings (MH) algorithm. In brief, Bayes’ theorem describes the relationship between the posterior distribution to be maximized and the known (or assumed) prior distribution

$$f\left( {\theta |y} \right) = \frac{{f\left( {y |\theta } \right)f\left( \theta \right)}}{f\left( y \right)} \propto f\left( {y |\theta } \right)f\left( \theta \right),$$

where represents the data likelihood function, $f\left( \theta \right)$ is our prior knowledge on *θ* and $f\left( y \right)$ is the probability of the data. Here, is constant, as the experimental measurements are known. The likelihood function estimates the goodness of fit of a model given the unknown parameter values. It captures the error, $\in$ , between the model predictions and the experimental data: $\in = y - {\mathcal{M}}\left( \theta \right)$ (both *y* and are vectors). Thus, the likelihood function is directly related to the error:We make the assumption that *y* is normally distributed with mean equal to zero and variance equal to $\sigma^{2}$. We can marginalize out the noise from $f\left( {y |\theta ,\sigma^{2} } \right)$ by assuming an inverse gamma distribution over $\sigma^{2}$ and integrating with respect to $\sigma^{2}$ to attain

$$f\left( {y|\theta } \right) = \int\limits_{0}^{\infty } {f\left( {y|\theta ,\sigma ^{2} } \right)f\left( {\sigma ^{2} } \right){\text{d}}\sigma ^{2} }$$

The density of the likelihood function is at its maximum when $y = {\mathcal{M}}\left( \theta \right)$ since is centered at zero. Therefore, maximizing the posterior density is equivalent to minimizing the error between the model prediction and the experimental data. We cannot solve for analytically since is a nonlinear operator, so we employ the Metropolis–Hastings (MH) algorithm^23^,^48^ to sample from the posterior distribution, which is the target distribution. The prior distribution remains fixed over all iterations while the proposal distribution re-centers around parameters $\theta^{*}$ that minimize the error between the model and the data.

Since this parameter estimation approach is probabilistic, we simulated the MH algorithm 50 independent times with a random initial guess for the parameter values. Within each independent fit, 10,000 iterations on the parameter values were performed to effectively sample from the posterior distribution for each parameter value. The first several thousand iterations of the MH algorithm serve to maximize the posterior density, thereby converging the initial estimate of the posterior distribution closer to the true posterior distribution. This is known as the burning-in phase. Once the algorithm converges, then each $\theta^{\left( i \right)}$ (for *i* sufficiently large) will be a sample from the posterior distribution. We discarded the first 9000 iterations, retaining the last 1000 iterations. We note that we do not make the assumption that the parameters have unique values. Rather, the Bayesian approach assumes that the parameters are random variables, and a distribution is imposed on them ($f\left( \theta \right)$, the prior distribution). Examining the posterior distribution provides an indication of whether the estimated parameters are identifiable, given the experimental data available for fitting. We show the posterior distribution of each parameter in Figs. S5–S7 for model structures 5, 7, and 8, respectively. These figures demonstrate that the parameters are well behaved: the distributions are unimodal, and the values lie within a tight range. In addition, we provide diagnostic information in the form of trace plots to further demonstrate that the parameters are identifiable (Figs. S8–S10 for model structures 5, 7, and 8, respectively).

For model structures 5 and 7, the best fit was taken to be the independent fit with the lowest median error within the last 1000 iterations. For model structure 8, the best fit was taken to be the independent fit with the second lowest median error within the last 1000 iterations since the independent fit with the lowest median error had fluctuations in parameter values within the last 1000 iterations.

We used the Akaike Information Criterion (AIC) to compare model structures with various combinations of regulatory mechanisms. For Table 1, AIC was calculated from median sum of squared error (SSE) values for each model structure as:

$$AIC = n \times \log \left( {\frac{\text{SSE}}{n}} \right) + 2k$$

where *n* is the number of data points, SSE is the median error, and *k* is the number of parameters used to fit the model.

To display the results for Fig. 6 and Figs. S3 and S4, the predicted time courses were simulated for each of the 1000 parameter sets from the posterior. The mean and standard deviation of the model predictions were quantified and shown. For dose response predictions (Fig. 7), the dose response data for rat primary beta cells was quantified using Plot Digitizer from Brelje *et al.* Fig. 13.^11^

## Electronic supplementary material

Below is the link to the electronic supplementary material.

Supplementary material 1 (XLSX 67 kb)

Supplementary material 2 (PDF 6778 kb)
