# Supplementary material for: Dynamic Regulation of JAK-STAT Signaling Through the Prolactin Receptor Predicted by Computational Modeling
Source: Cell Mol Bioeng. 2020 Sep 8;14(1):15–30. doi: 10.1007/s12195-020-00647-8 (PMC7878662; doi:10.1007/s12195-020-00647-8)

Supplemental File S2

Dynamic regulation of JAK-STAT signaling through the prolactin receptor predicted by computational modeling

Ryland D. Mortlock[1], Senta K. Georgia[2] Stacey D. Finley[1,3,4*]

[1]Mork Family Department of Chemical Engineering and Materials Science, University of Southern California, Los Angeles, CA
[2]Departments of Pediatrics and Stem Cell Biology and Regenerative Medicine, Keck School of Medicine, University of Southern California, Los Angeles, CA
[3]Department of Biomedical Engineering, University of Southern California, Los Angeles, CA
[4]Department of Biological Sciences, University of Southern California, Los Angeles, CA

Figure S1: Decision Tree for Shape Classification. All 800,000 Monte Carlo simulations were grouped into eight mutually exclusive shapes based on the prediction for total amount of pSTAT5, which is abbreviated as pStat in the figure. The abbreviation “pks” describes the vector of local maxima in pStat model predictions returned by Matlab’s *findpeaks* function. The abbreviation “loc” describes the time at which the local maxima occur. Similarly, the abbreviation “vl_loc” describes the time values corresponding to local minima in the pStat predictions. The decision tree reads from left to right along the gold line, meaning that once a decision is made that brings a shape “off of the gold line”, subsequent decisions are not made on that simulation. Thus, the simulations matching the desired shape are those which have not been filtered out by any of the logical statements shown in the decision tree.

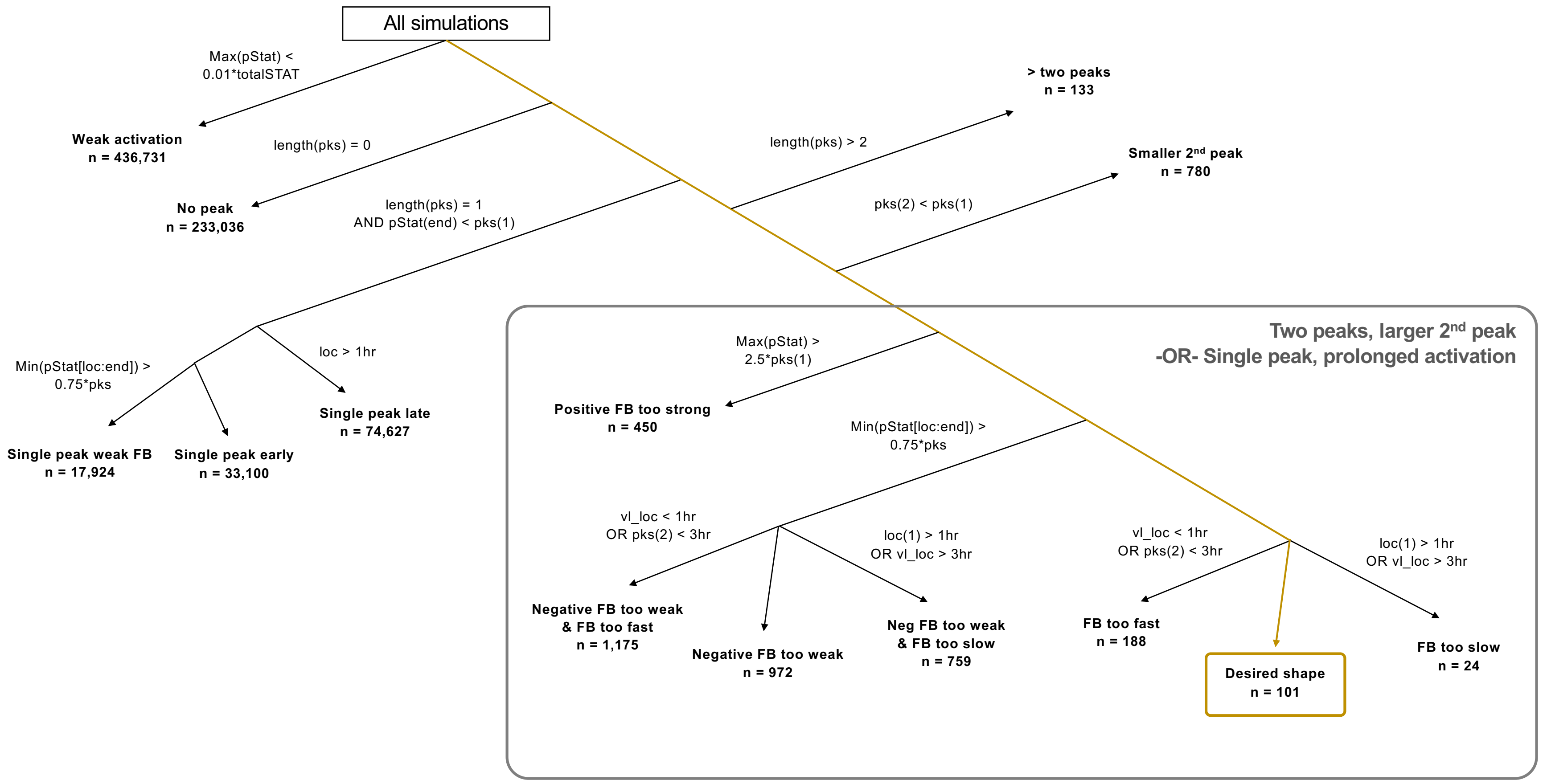

Figure S2: Robustness of Monte Carlo Simulations. For each model structure which had some simulations matching the desired shape (Fig. 4), we added a specified noise factor (x) to each parameter value, representing ± x% of the parameter value. We repeated 100 times at each value of the noise factor and quantified what percentage of simulations still matched the desired qualitative shape of STAT5 activation. **(A)** Results are for each model structure. **(B)** Results for each model structure with log-scale on the y-axis.

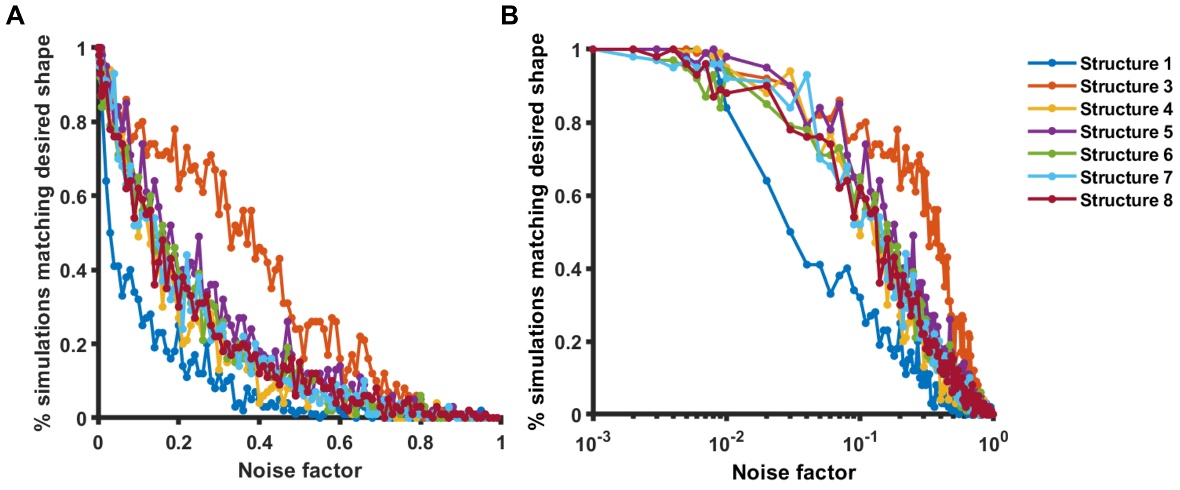

Figures S3: Model Calibration for Model Structure 5. Lines show mean value of model predictions with shading showing the standard. The letters in the top left correspond to which of the regulatory modules from Fig. 1 are included. Squares show experimental data points from Brelje *et al.* for panels A, B, C, and D or from Fujinaka *et al.* for panel E. Error bars are included for experimental data points that had error bars shown in the previously published work. All experimental data is for INS-1 cells treated with PRL at 200 ng/mL. *Dark blue,* STAT5A; *light blue,* STAT5B in panel D.

a b -

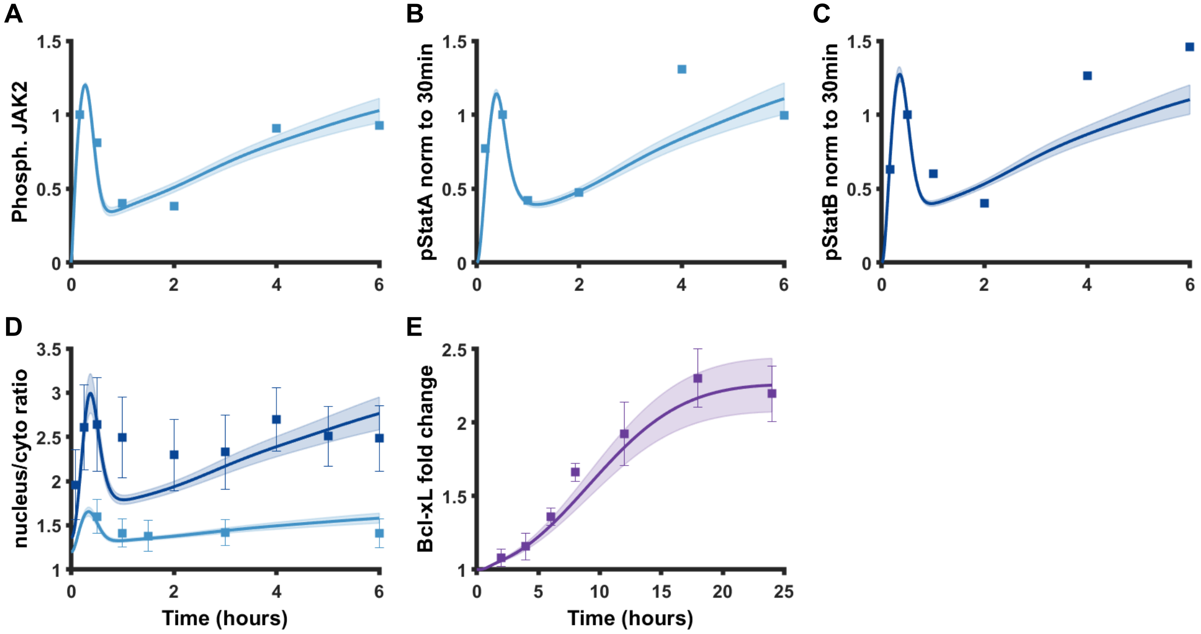

Figures S4: Model Calibration for Model Structure 7. Lines show mean value of model predictions with shading showing the standard. The letters in the top left correspond to which of the regulatory modules from Fig. 1 are included. Squares show experimental data points from Brelje *et al.* for panels A, B, C, and D or from Fujinaka *et al.* for panel E. Error bars are included for experimental data points that had error bars shown in the previously published work. All experimental data is for INS-1 cells treated with PRL at 200 ng/mL. *Dark blue,* STAT5A; *light blue,* STAT5B in panel D.

- b c

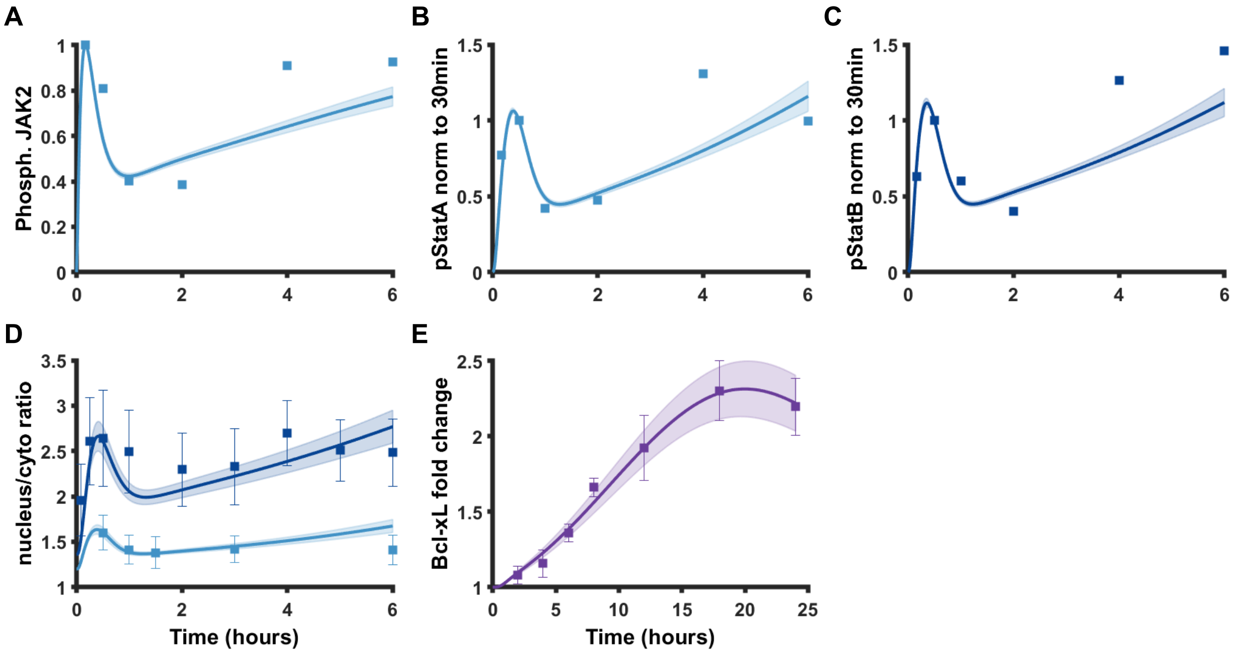

Figure S5: Parameter Value Distributions from Best-Fit Likelihood Estimation for Structure 5. Histograms showing the posterior distribution of each parameter value from the last 1,000 iterations of Bayesian estimation, shown for the independent fit with the second lowest error for structure 5 (see Methods section).

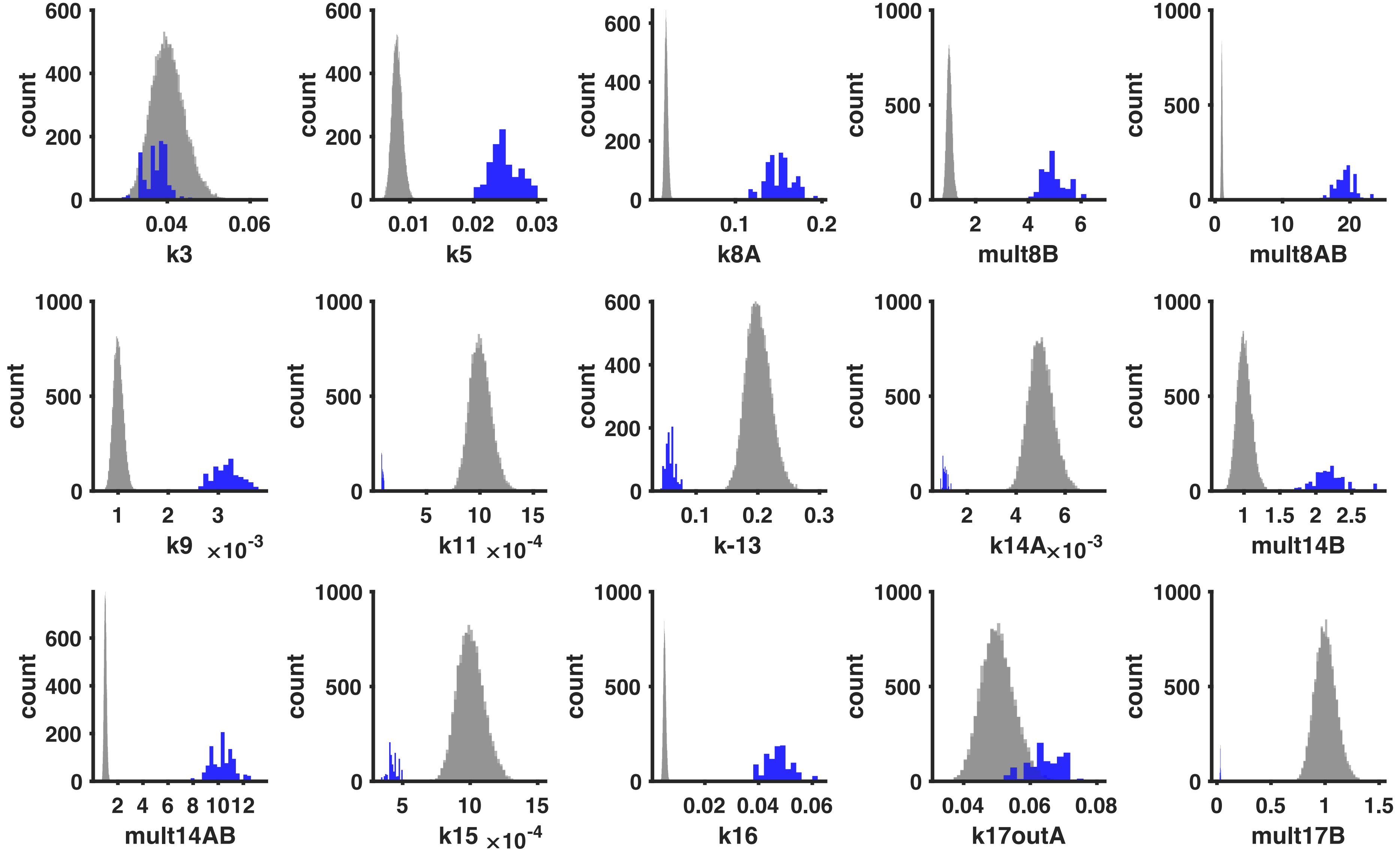

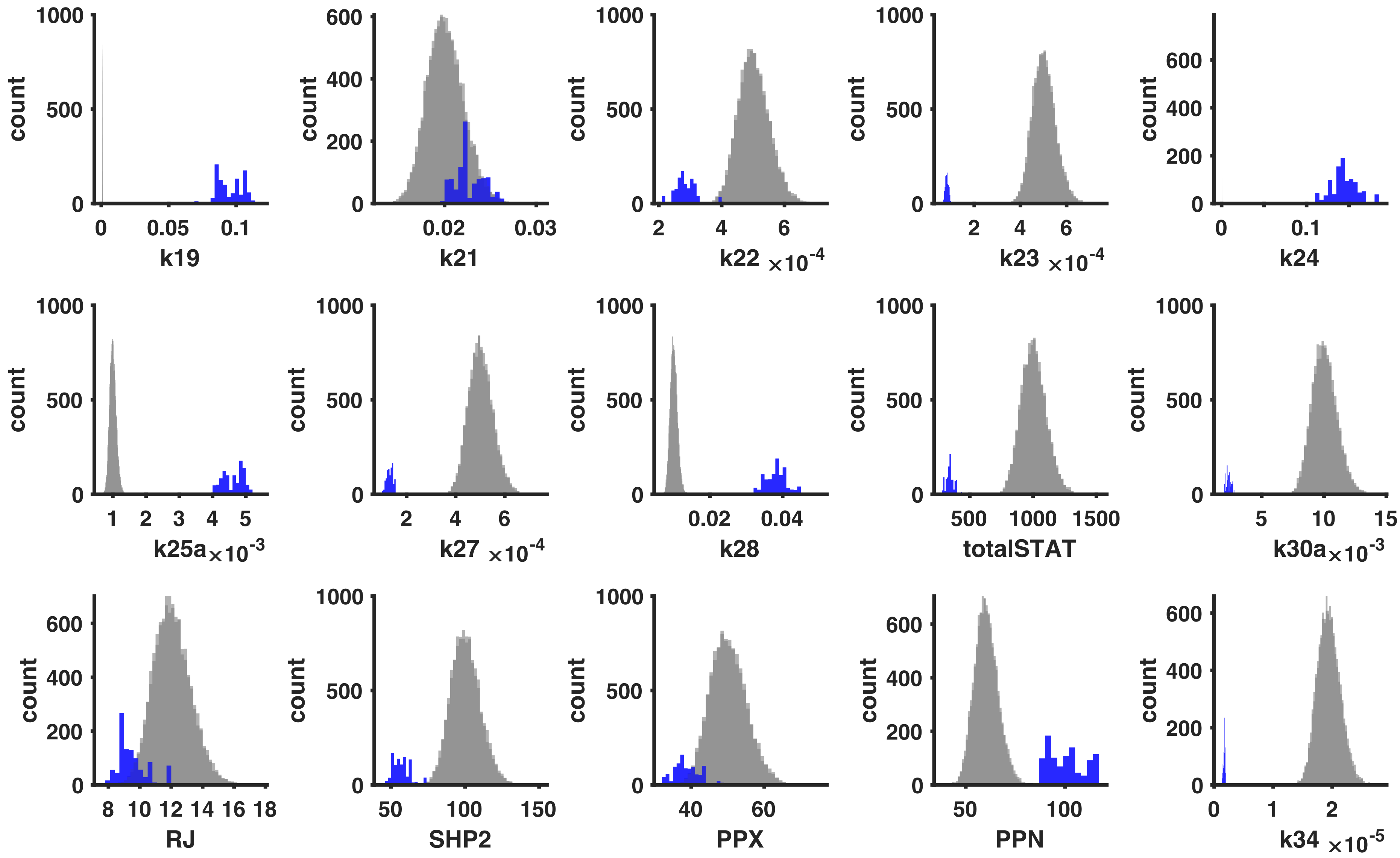
count
k19
k21
k22 ×10^-4
k23 ×10^-4
k24
k25a ×10^-3
k27 ×10^-4
k28
totalSTAT
k30a ×10^-3
RJ
SHP2
PPX
PPN
k34 ×10^-5

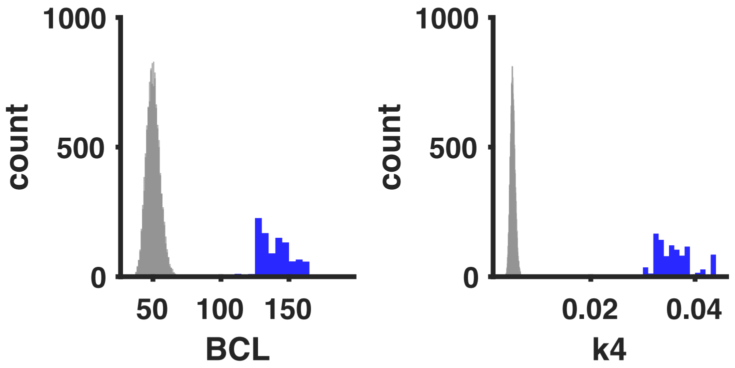

1000
500
0
count
50
100
150
BCL
1000
500
0
count
0.02
0.04
k4

Figure S6: Parameter Value Distributions from Best-Fit Likelihood Estimation for Structure 7. Histograms showing the posterior distribution of each parameter value from the last 1,000 iterations of Bayesian estimation, shown for the independent fit with the lowest error for structure 7.

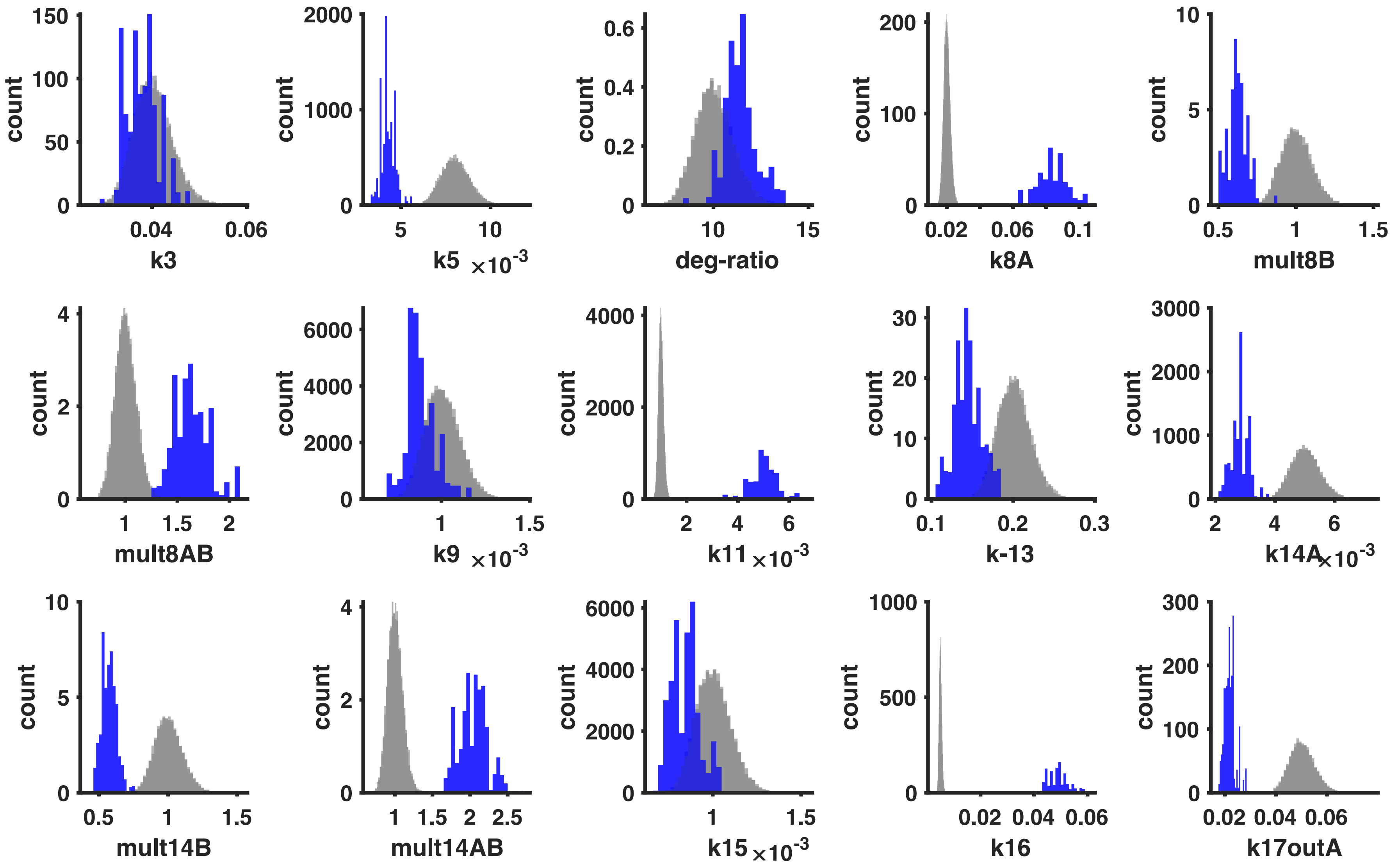

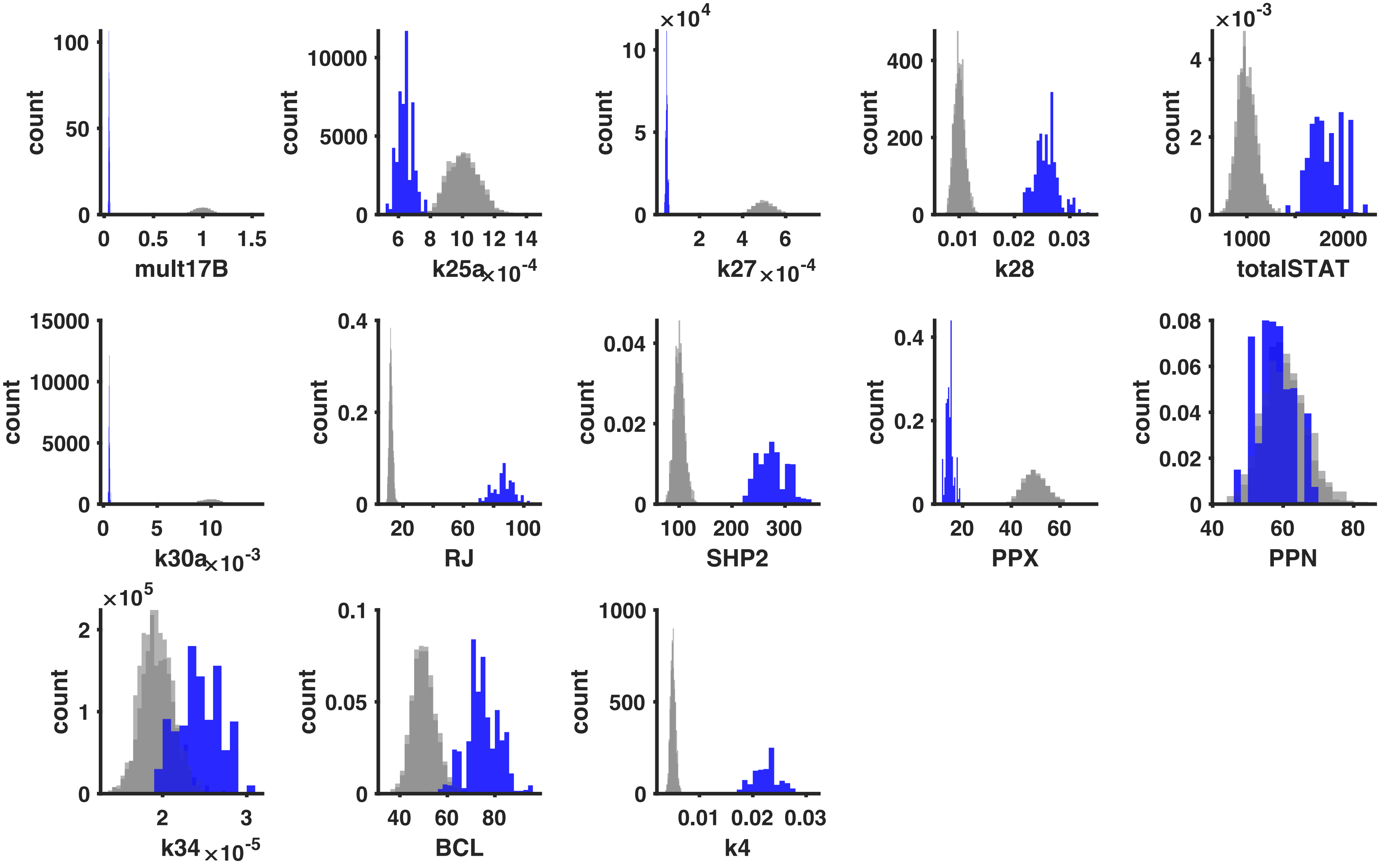
count
mult17B
0 0.5 1 1.5
0 50 100
k25a
×10^-4
6 8 10 12 14
0 5000 10000
k27
×10^-4
×10^4
2 4 6
0 5 10
k28
0.01 0.02 0.03
0 200 400
totalSTAT
×10^-3
1000 2000
0 2 4
k30a
×10^-3
0 5 10
0 5000 10000 15000
RJ
20 60 100
0 0.2 0.4
SHP2
100 200 300
0 0.02 0.04
PPX
20 40 60
0 0.2 0.4
PPN
40 60 80
0 0.02 0.04 0.06 0.08
k34
×10^-5
×10^5
2 3
0 1 2
BCL
40 60 80
0 0.05 0.1
k4
0.01 0.02 0.03
0 500 1000

Figure S7: Parameter Value Distributions from Best-Fit Likelihood Estimation for Structure 8. Histograms showing the posterior distribution of each parameter value from the last 1,000 iterations of Bayesian estimation, shown for the independent fit with the lowest error for structure 8.

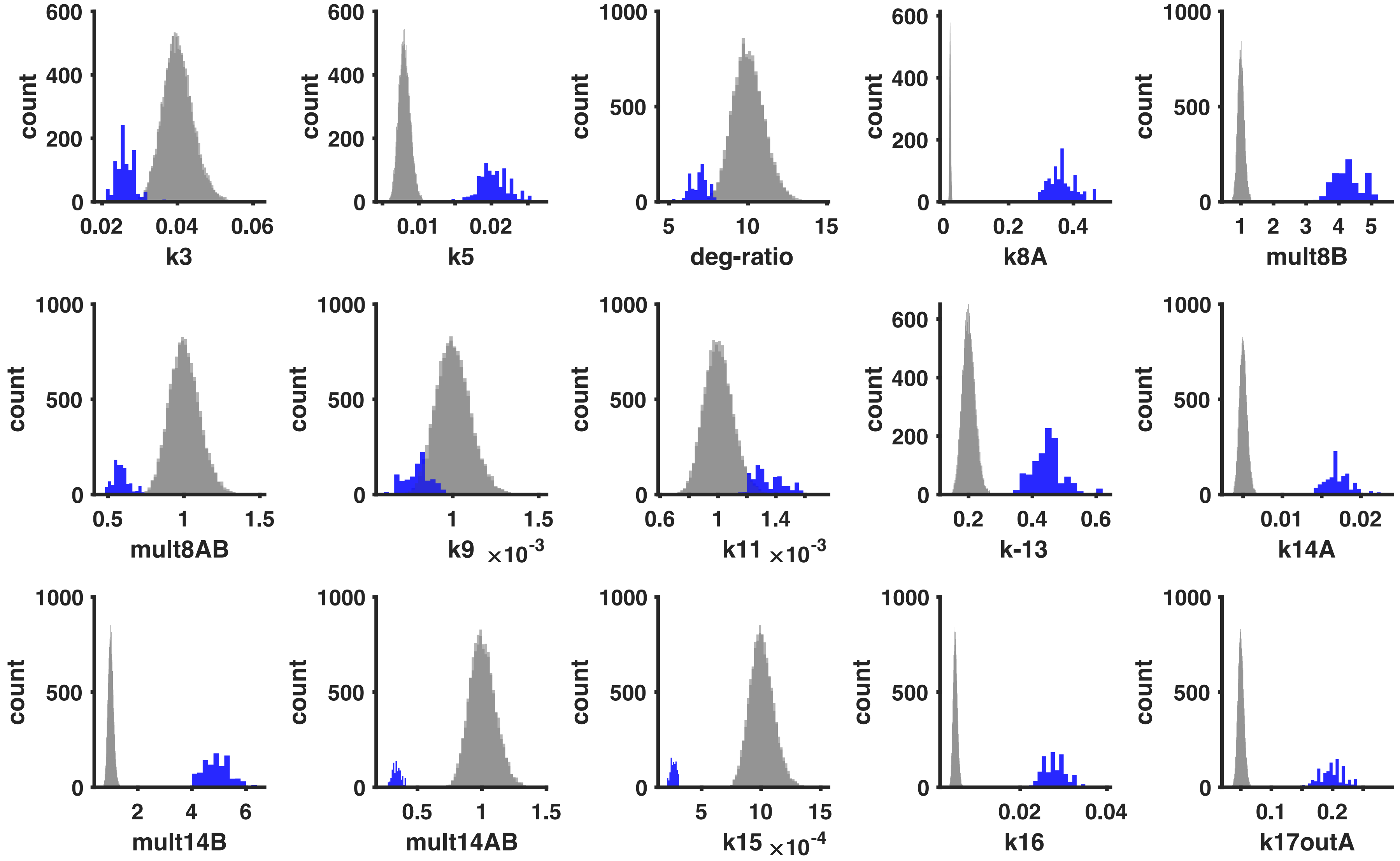

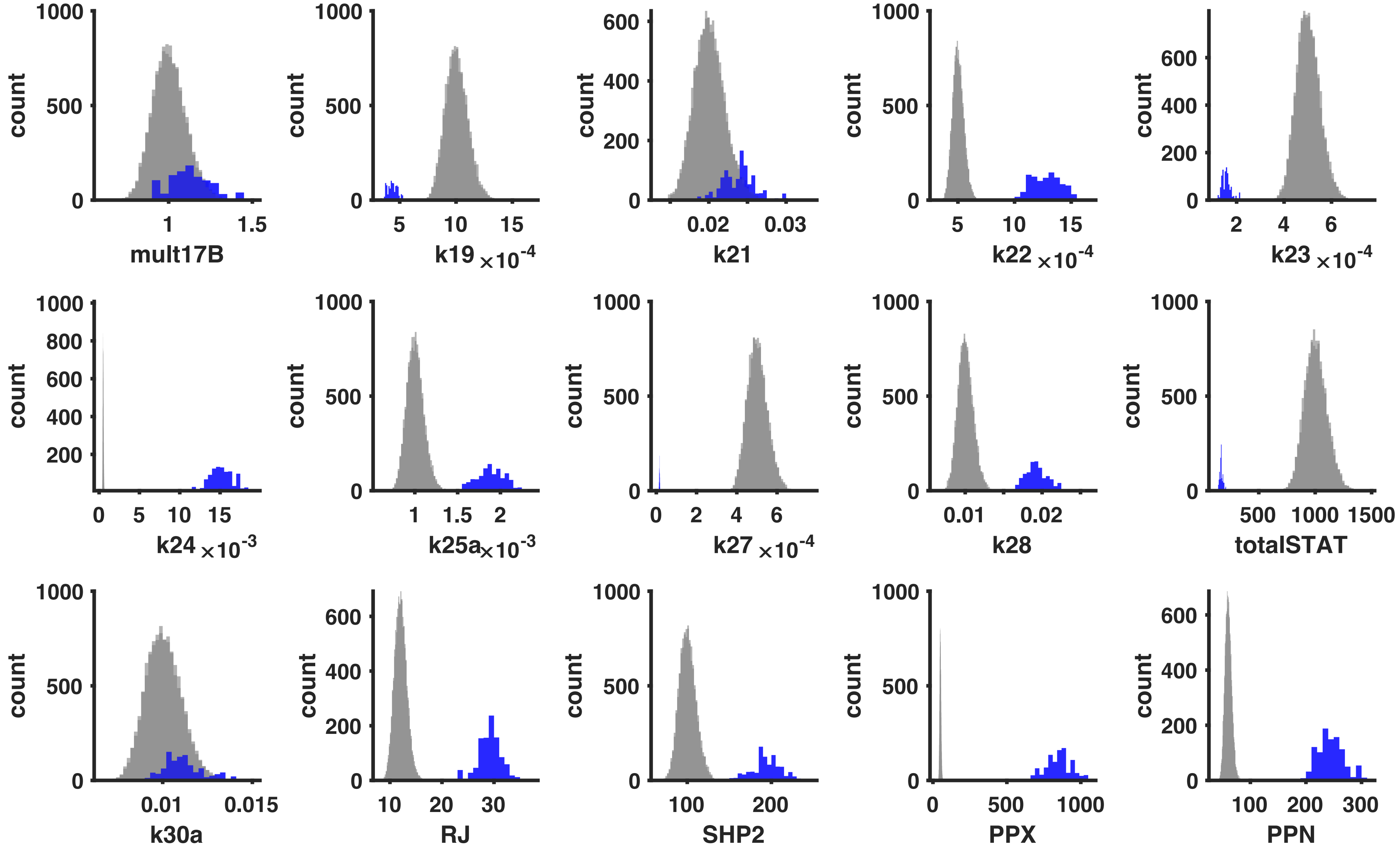

count
mult17B
k19 ×10^-4
k21
k22 ×10^-4
k23 ×10^-4
k24 ×10^-3
k25a ×10^-3
k27 ×10^-4
k28
totalSTAT
k30a
RJ
SHP2
PPX
PPN

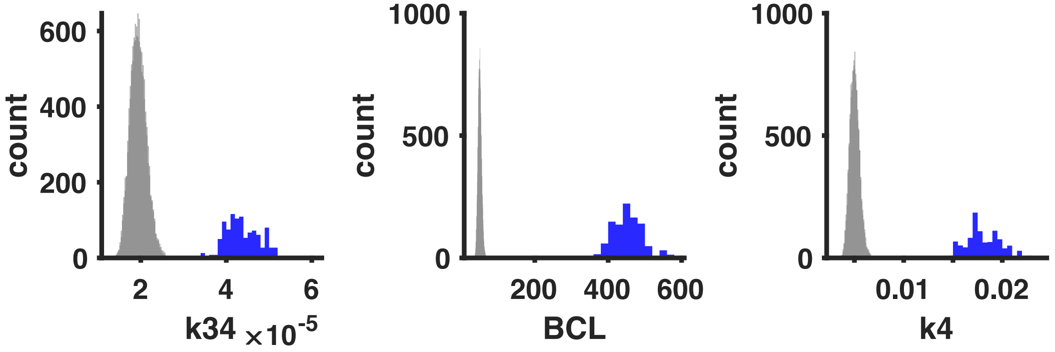

count
600
400
200
0
2
4
6
k34 ×10^-5
count
1000
500
0
200
400
600
BCL
count
1000
500
0
0.01
0.02
k4

Figure S8: Parameter Trace Plots for Structure 5. Trace plots showing the value of each parameter over the course of 10,000 iterations of Bayesian estimation, shown for the independent fit with the lowest error for structure 5.

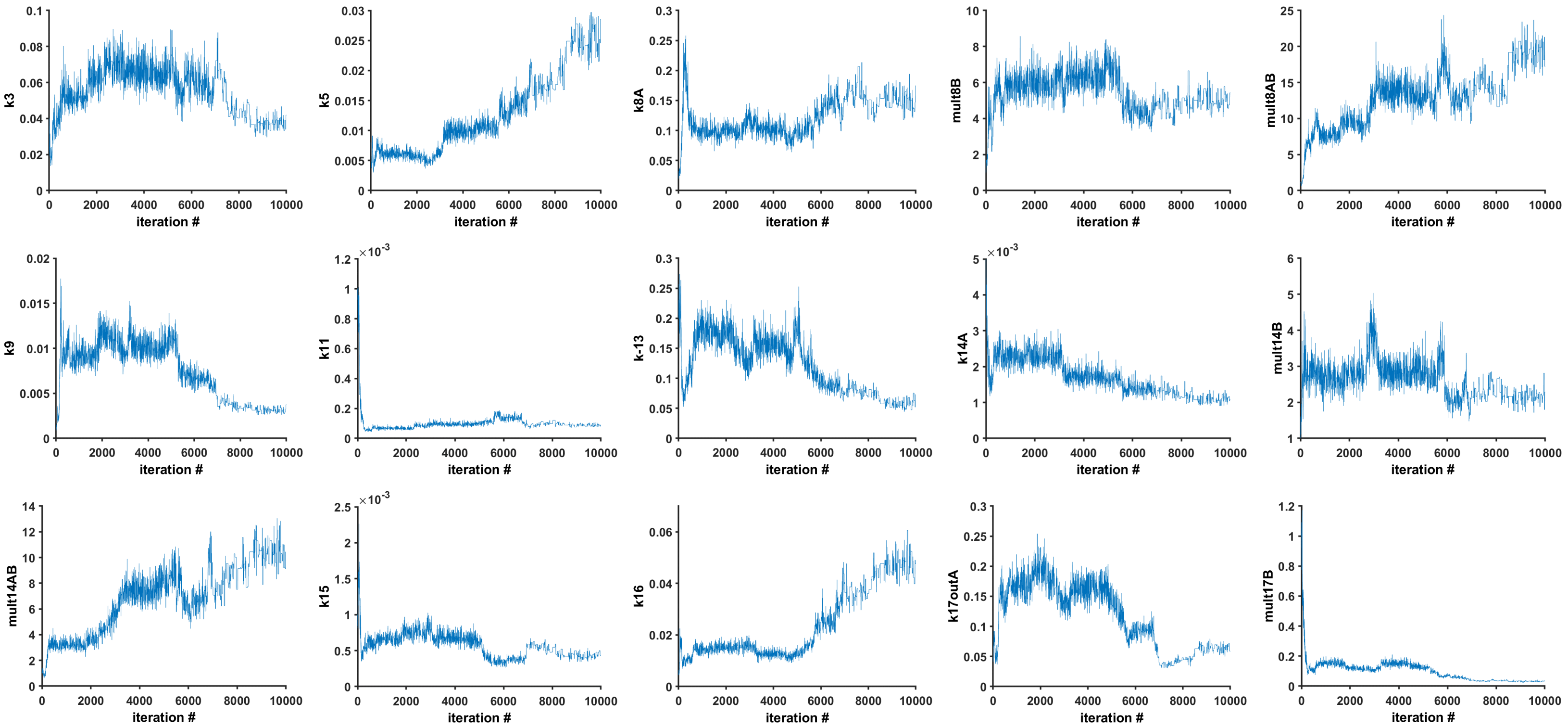

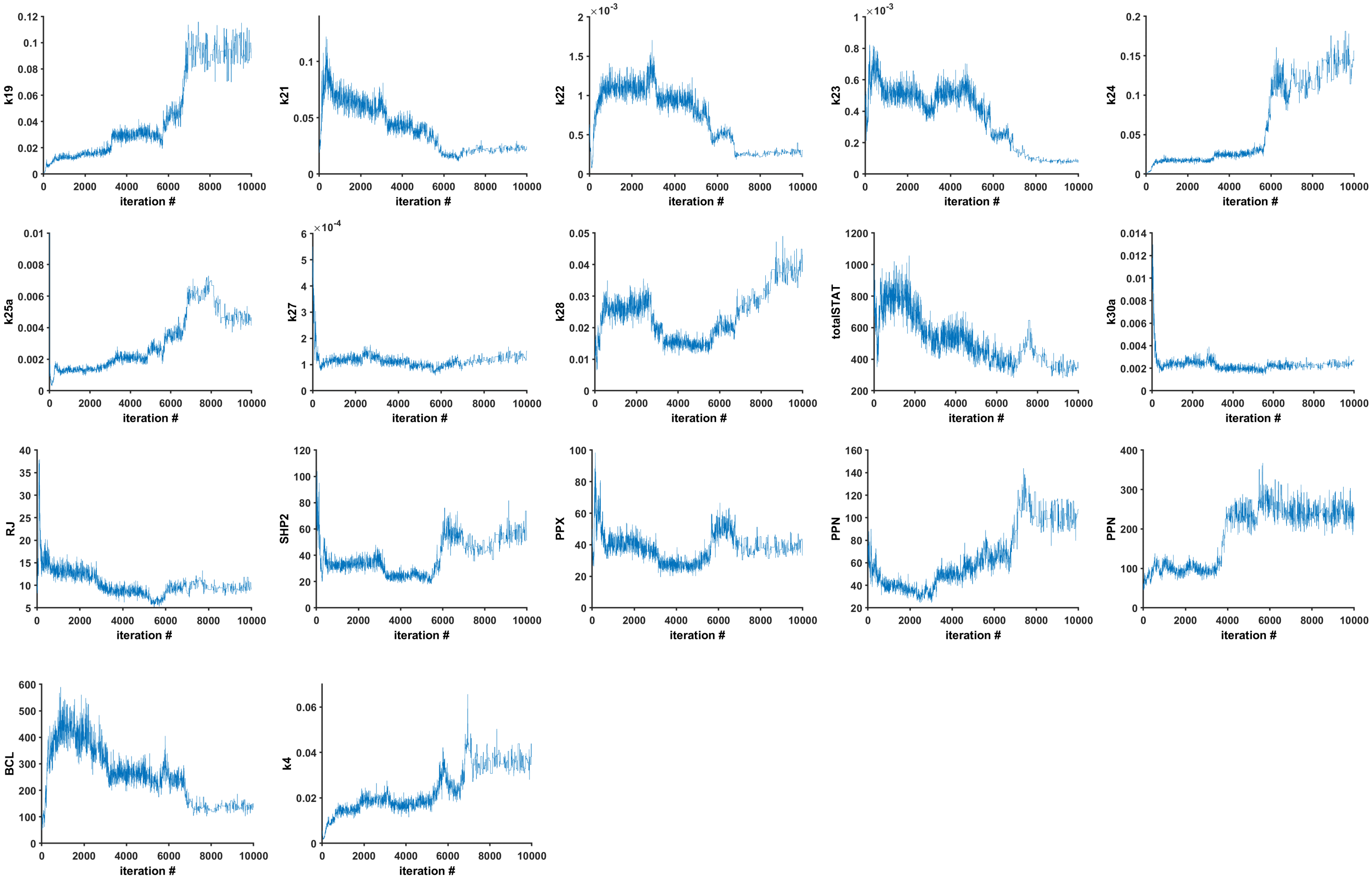
k19
k21
k22
k23
k24
k25a
k27
k28
totalSTAT
k30a
RJ
SHP2
PPX
PPN
PPN
BCL
k4
iteration #

Figure S9: Parameter Trace Plots for Structure 7. Trace plots showing the value of each parameter over the course of 10,000 iterations of Bayesian estimation, shown for the independent fit with the lowest error for structure 7.

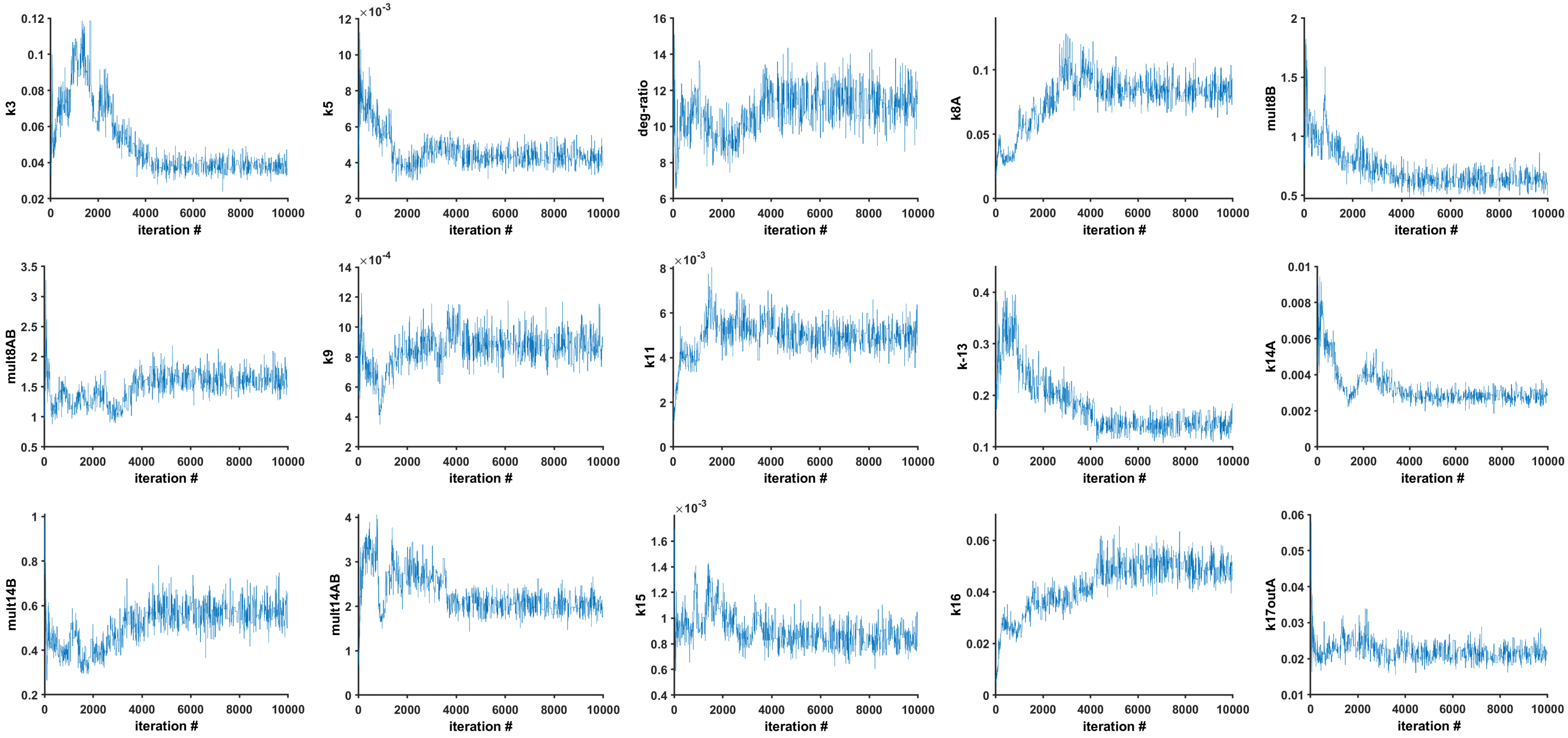

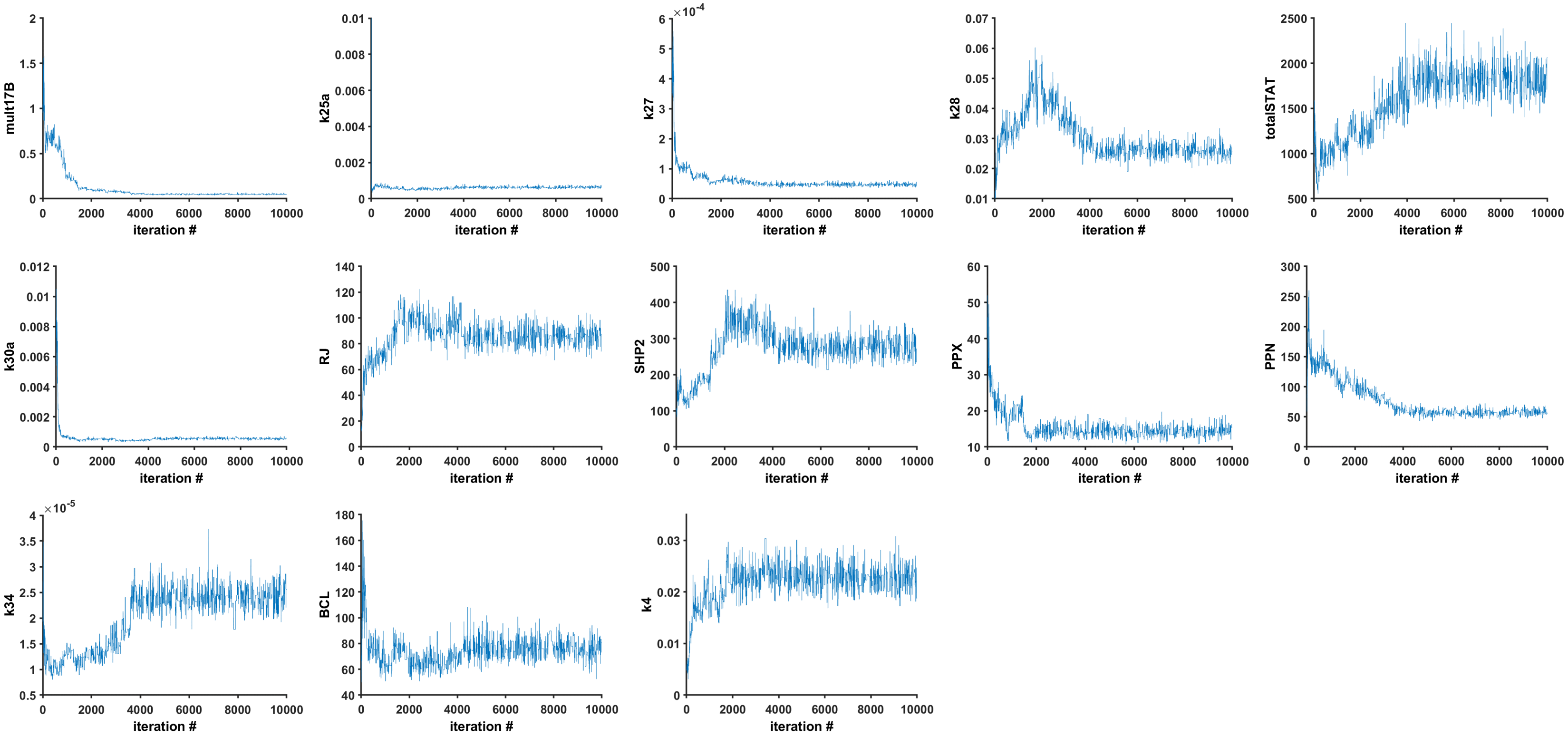
mult17B
k25a
k27
$\times 10^{-4}$
k28
totalSTAT
k30a
RJ
SHP2
PPX
PPN
k34
$\times 10^{-5}$
BCL
k4
iteration #

Figure S10: Parameter Trace Plots for Structure 8. Trace plots showing the value of each parameter over the course of 10,000 iterations of Bayesian estimation, shown for the independent fit with the second lowest error for structure 8 (see Methods section).

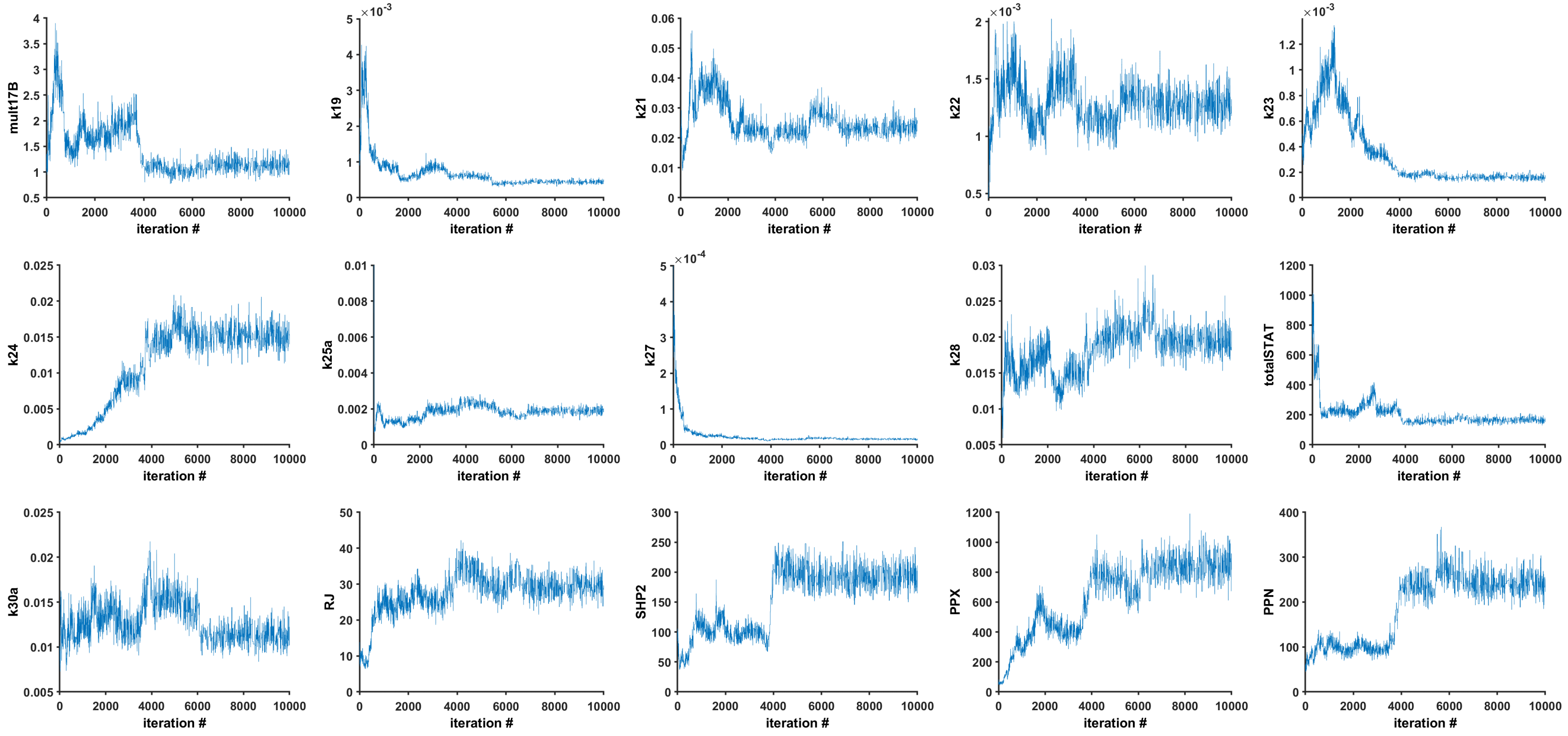

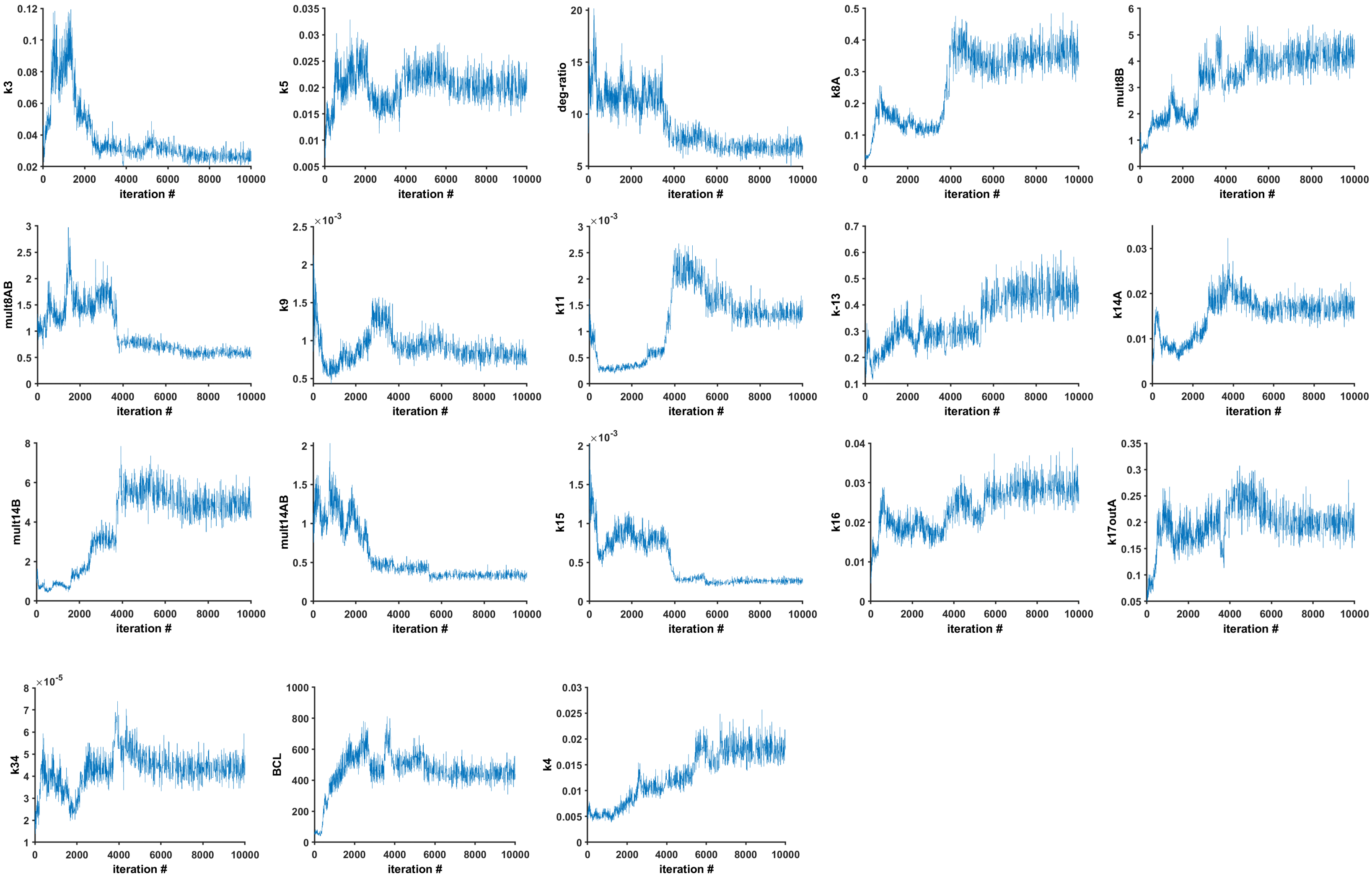

k3
k5
deg-ratio
k8A
mult8B
mult8AB
k9
$\times 10^{-3}$
k11
$\times 10^{-3}$
k-13
k14A
mult14B
mult14AB
k15
$\times 10^{-3}$
k16
k17outA
k34
$\times 10^{-5}$
BCL
k4
iteration #

Figure S11: Error Values for 50 Independent Fits for Each Model Structure. Histogram showing the distribution of error values for 50 independent fits (left). The error value plotted against the number of fits, ranked by lowest error (right). **(A)** Model structure 5, **(B)** Module structure 7, **(C)** Model structure 8.

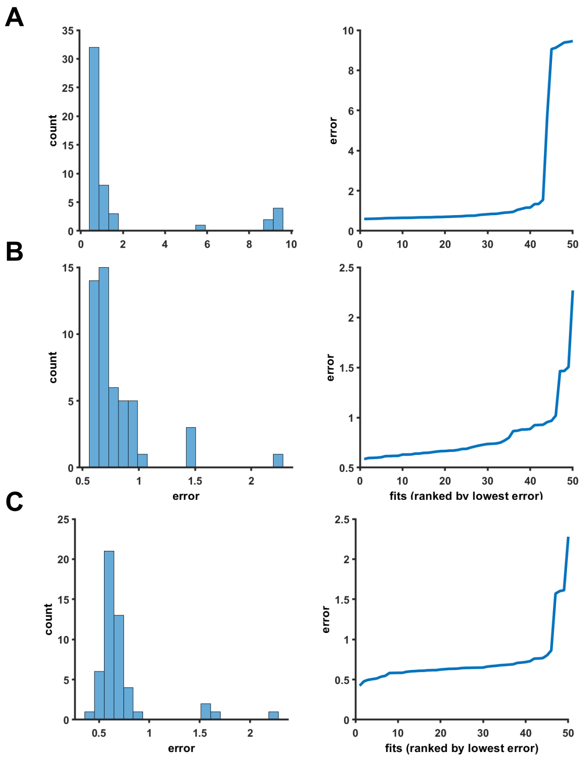

Figure S12: Effect of Varying Single Parameters on Model Predictions. Each of the parameters (**Panel A,** *k2*; **Panel B,** *k12*) or initial values (**Panel C,** *PPX*; **Panel D,** *RJ*) were varied one order of magnitude smaller and larger than the fitted value in order to evaluate how perturbations to single parameter values affect model predictions. Lines indicate the mean model prediction from the 1,000 posterior parameter sets. STAT5A predictions are shown separately from STAT5B for ease of viewing. *Dark blue,* STAT5A; *light blue,* STAT5B; *purple,* Bcl-xL. Note the different y-axis limits for Panel D.

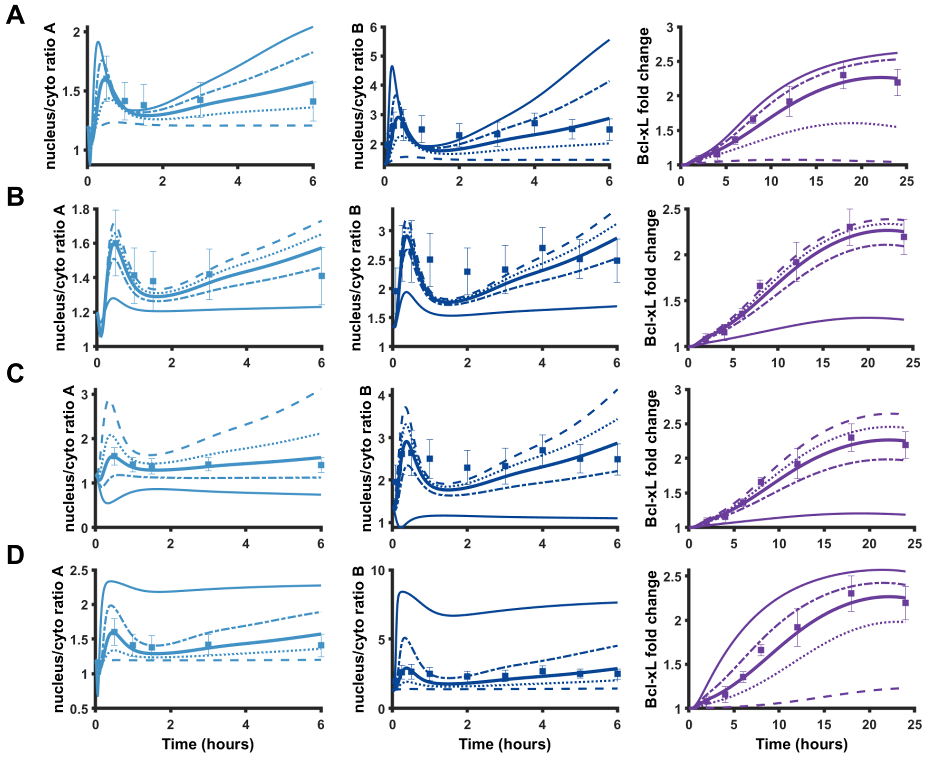

Supplement: Supplementary file 2 — Supplementary material 2 (PDF 6778 kb) [file 12195_2020_647_MOESM2_ESM.pdf]
